# Farnesoid X Receptor Signaling Shapes the Gut Microbiota and Controls Hepatic Lipid Metabolism

**DOI:** 10.1128/mSystems.00070-16

**Published:** 2016-10-11

**Authors:** Limin Zhang, Cen Xie, Robert G. Nichols, Siu H. J. Chan, Changtao Jiang, Ruixin Hao, Philip B. Smith, Jingwei Cai, Margaret N. Simons, Emmanuel Hatzakis, Costas D. Maranas, Frank J. Gonzalez, Andrew D. Patterson

**Affiliations:** a Center for Molecular Toxicology and Carcinogenesis, Department of Veterinary and Biomedical Sciences, The Pennsylvania State University, University Park, Pennsylvania, USA; b CAS Key Laboratory of Magnetic Resonance in Biological Systems, State Key Laboratory of Magnetic Resonance and Atomic and Molecular Physics, National Centre for Magnetic Resonance in Wuhan, Wuhan Institute of Physics and Mathematics, Chinese Academy of Sciences, Wuhan, China; c Laboratory of Metabolism, National Cancer Institute, National Institutes of Health, Bethesda, Maryland, USA; d Department of Chemical Engineering, The Pennsylvania State University, University Park, Pennsylvania, USA; e Department of Physiology and Pathophysiology, School of Basic Medical Sciences, Peking University, and Key Laboratory of Molecular Cardiovascular Science, Ministry of Education, Beijing, China; f Huck Institutes of the Life Sciences, The Pennsylvania State University, University Park, Pennsylvania, USA; g Department of Chemistry, The Pennsylvania State University, University Park, Pennsylvania, USA; h Department of Food Science and Technology, The Ohio State University, Columbus, Ohio, USA; Argonne National Laboratory

**Keywords:** bile acid, farnesoid X receptor, genome-scale metabolic models, gut microbiota, metabolomics, nonalcoholic fatty liver disease

## Abstract

The gut microbiota modulates obesity and associated metabolic phenotypes in part through intestinal farnesoid X receptor (FXR) signaling. Glycine-β-muricholic acid (Gly-MCA), an intestinal FXR antagonist, has been reported to prevent or reverse high-fat diet (HFD)-induced and genetic obesity, insulin resistance, and fatty liver; however, the mechanism by which these phenotypes are improved is not fully understood. The current study investigated the influence of FXR activity on the gut microbiota community structure and function and its impact on hepatic lipid metabolism. Predictions about the metabolic contribution of the gut microbiota to the host were made using 16S rRNA-based PICRUSt (**p**hylogenetic **i**nvestigation of **c**ommunities by **r**econstruction of **u**nobserved **st**ates), then validated using ^1^H nuclear magnetic resonance-based metabolomics, and results were summarized by using genome-scale metabolic models. Oral Gly-MCA administration altered the gut microbial community structure, notably reducing the ratio of *Firmicutes* to *Bacteroidetes* and its PICRUSt-predicted metabolic function, including reduced production of short-chain fatty acids (substrates for hepatic gluconeogenesis and *de novo* lipogenesis) in the ceca of HFD-fed mice. Metabolic improvement was intestinal FXR dependent, as revealed by the lack of changes in HFD-fed intestine-specific *Fxr*-null (*Fxr*^ΔIE^) mice treated with Gly-MCA. Integrative analyses based on genome-scale metabolic models demonstrated an important link between *Lactobacillus* and *Clostridia* bile salt hydrolase activity and bacterial fermentation. Hepatic metabolite levels after Gly-MCA treatment correlated with altered levels of gut bacterial species. In conclusion, modulation of the gut microbiota by inhibition of intestinal FXR signaling alters host liver lipid metabolism and improves obesity-related metabolic dysfunction.

**IMPORTANCE** The farnesoid X receptor (FXR) plays an important role in mediating the dialog between the host and gut microbiota, particularly through modulation of enterohepatic circulation of bile acids. Mounting evidence suggests that genetic ablation of *Fxr* in the gut or gut-restricted chemical antagonism of the FXR promotes beneficial health effects, including the prevention of nonalcoholic fatty liver disease in rodent models. However, questions remain unanswered, including whether modulation of FXR activity plays a role in shaping the gut microbiota community structure and function and what metabolic pathways of the gut microbiota contribute in an FXR-dependent manner to the host phenotype. In this report, new insights are gained into the metabolic contribution of the gut microbiota to the metabolic phenotypes, including establishing a link between FXR antagonism, bacterial bile salt hydrolase activity, and fermentation. Multiple approaches, including unique mouse models as well as metabolomics and genome-scale metabolic models, were employed to confirm these results.

## Abstract (precis)

The farnesoid X receptor (FXR) plays an important role in mediating the dialog between the host and gut microbiota, particularly through modulation of enterohepatic circulation of bile acids. Mounting evidence suggests that genetic ablation of *Fxr* in the gut or gut-restricted chemical antagonism of the FXR promotes beneficial health effects, including the prevention of nonalcoholic fatty liver disease in rodent models. However, questions remain unanswered, including whether modulation of FXR activity plays a role in shaping the gut microbiota community structure and function and what metabolic pathways of the gut microbiota contribute in an FXR-dependent manner to the host phenotype. In this report, new insights are gained into the metabolic contribution of the gut microbiota to the metabolic phenotypes, including establishing a link between FXR antagonism, bacterial bile salt hydrolase activity, and fermentation. Multiple approaches, including unique mouse models as well as metabolomics and genome-scale metabolic models, were employed to confirm these results.

## INTRODUCTION

The increased prevalence of obesity and its related metabolic disorders continues to be a major global health issue due to multiple factors, including genetics, lifestyle, environmental chemical exposure, and diet (1–3). Obesity is considered a major risk factor for chronic diseases such as type 2 diabetes mellitus, atherosclerosis, and cancer (4, 5). From a metabolism perspective, obesity is the result of an imbalance of energy intake and energy expenditure, thus leading to excess fat storage in liver and adipose tissue, and subsequently it can promote multiple metabolic disorders (6, 7).

Recent evidence suggests that modulation of farnesoid X receptor (FXR) signaling has beneficial effects on the development of obesity (8–11). FXR is a bile acid-activated nuclear receptor that regulates the homeostasis of bile acids, lipids, and glucose (12–14). Endogenous ligands of FXR include bile acids such as cholic acid (CA), chenodeoxycholic acid (CDCA), deoxycholic acid (DCA), lithocholic acid (LCA), and ursodeoxycholic acid (UDCA) (14, 15). UDCA is used to treat human liver diseases, such as nonalcoholic fatty liver disease (NAFLD) and nonalcoholic steatohepatitis (NASH) (16). Further, UDCA was found to improve NASH, insulin resistance, and high-fat diet (HFD)-induced obesity through suppression of FXR signaling, which is manifested by a significant reduction of FXR and fibroblast growth factor 19 (FGF19) levels coupled with elevation of cholesterol 7α-hydroxylase (CYP7A1) expression in the intestine (17). Interestingly, tauro-β-muricholic acid (T-β-MCA) was also identified as a naturally occurring FXR antagonist that inhibits FXR signaling *in vivo* in mouse intestine (9, 18). Previous studies showed that tempol, an antioxidant, and antibiotic treatments resulted in reduction of the genus *Lactobacillus*, thus improving obesity, NAFLD, and insulin resistance via inhibition of intestinal FXR signaling (9, 11). However, T-β-MCA is rapidly metabolized in the ileum by bacterial bile salt hydrolase (BSH) through deconjugation, yielding β-MCA and taurine (19–21). Therefore, a new high-affinity intestinal FXR antagonist, glycine-β-muricholic acid (Gly-MCA), was designed that is structurally and functionally similar to T-β-MCA and that demonstrated stability in the gut by its resistance to hydrolysis by BSH. Gly-MCA improved HFD-induced obesity and insulin resistance (11); however, the underlying mechanisms by which Gly-MCA alters the gut microbiota population and its impact on host metabolism remain largely undetermined.

In the current study, a combination of 16S rRNA gene sequencing, ^1^H nuclear magnetic resonance (NMR)-based metabolomics, and genome-scale metabolic models was used to investigate the alteration of the gut microbiota and host metabolome in HFD-fed mice treated with Gly-MCA. Intestine-specific *Fxr*-null (*Fxr*^ΔIE^) mice fed an HFD were also employed to explore the mechanism by which inhibition of FXR signaling improves obesity-related metabolic disorders. In addition, the correlation between the gut microbiome and host metabolome under Gly-MCA-treated conditions was analyzed with the goal of identifying a specific host-microbiota signaling axis that contributes to metabolic disorders, including obesity and NAFLD. This study provides new evidence that Gly-MCA has beneficial effects on obesity through modulation of the gut microbiota and inhibition of intestinal FXR signaling.

## RESULTS

### Gly-MCA modulates gut microbiota composition and related functional pathways.

**FIG 1:**
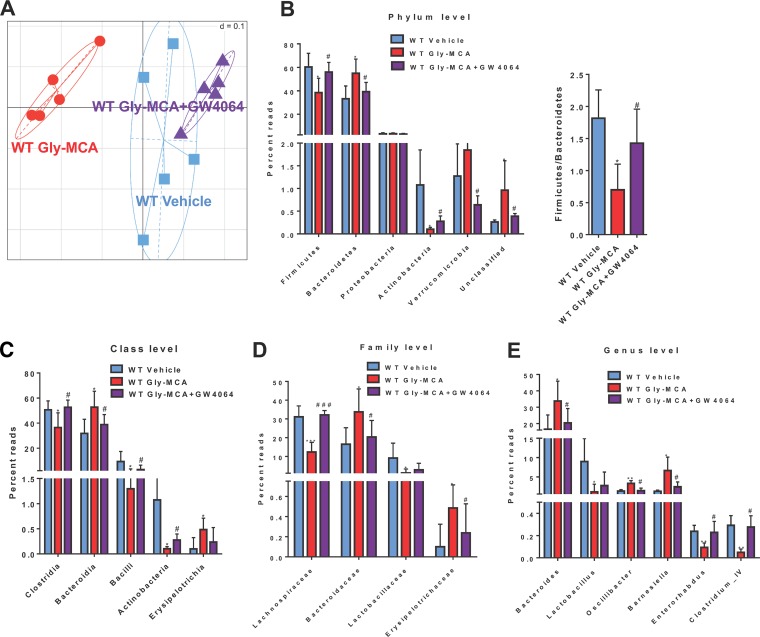
Gly-MCA alters the gut microbiota population and composition. HFD-fed mice were treated for 5 weeks with Gly-MCA (10 mg/kg). (A) Generalized Unifrac analysis of the total population of the gut microbiome of cecal contents from vehicle- and Gly-MCA-treated mice and mice treated with Gly-MCA plus GW4064. (B) 16S rRNA gene sequencing analysis at the phylum level of the cecal contents and ratio of *Firmicutes* to *Bacteroidetes*. (C to E) 16S rRNA gene sequencing analysis at the class (C), family (D) and genus (E) levels in the cecal contents. Data are presented as means ± SD (*n* = 5 per group). *, *P* < 0.05; **, *P* < 0.01 (compared with vehicle treatment). #, *P* < 0.05; ##, *P* < 0.01 (compared with Gly-MCA treatment of HFD-fed mice). Data were analyzed with a one-way ANOVA with Tukey’s correction.

Emerging evidence suggests that the intestinal microbiota may play a pivotal role in the development of obesity (22). Here, generalized Unifrac analysis of 16S rRNA gene sequencing results revealed distinct clustering of cecal communities isolated from vehicle- and Gly-MCA-treated mice fed an HFD. Changes in the composition of the gut microbiota induced by Gly-MCA were noted and revealed that the cecal community structure in HFD-fed mice is altered following Gly-MCA treatment (Fig. 1A). However, the treatment group that received GW4064 (a potent FXR agonist) plus Gly-MCA more closely resembled the vehicle-treated mice than Gly-MCA-treated mice, thus suggesting that activation of intestinal FXR by GW4064 reversed the changes in the gut microbiota in the Gly-MCA-treated HFD-fed mice (Fig. 1A). The separation of samples in the generalized UniFrac plot was likely due to the significant phylum-level shifts from *Firmicutes* (fold change of 1.57) to *Bacteroidetes* (fold change of 1.99), with a reduction of the *Firmicutes*/*Bacteroidetes* ratio observed in mouse cecum after Gly-MCA treatment (Fig. 1B). Gly-MCA treatment was also associated with decreased phylum levels of *Actinobacteria*, with a fold change of 11.47 (Fig. 1B; see also Fig. S1 in the supplemental material). Furthermore, increases were observed in the classes *Bacteroidia* and *Erysipelotrichia*, while decreases were observed in the classes *Clostridia*, *Actinobacteria*, and *Bacilli* in Gly-MCA-treated HFD-fed mice (Fig. 1C). In addition, Gly-MCA caused drastic decreases of the levels of the families *Lachnospiraceae* and *Lactobacillaceae* of the phylum *Firmicutes*. Gly-MCA also caused a significant elevation of *Bacteroidaceae*, *Erysipelotrichaceae*, and *Streptococcaceae* in the phylum *Bacteroidetes* (Fig. 1D). At the genus level, Gly-MCA-treated HFD-fed mouse cecal contents were abundant in *Bacteroides*, *Oscillibacter*, *Barnesiella*, and *Streptococcus* but depleted in *Lactobacillus*, *Enterorhabdus* and *Clostridium* cluster IV in comparison with the vehicle-treated HFD-fed mice (Fig. 1E; see also Fig. S1). Interestingly, all the significantly changed bacteria were mostly reversed by the combination of Gly-MCA and GW4064 treatment (Fig. 1B to E). These results suggest that Gly-MCA treatment modulates the gut microbiota community in the cecal contents of HFD-fed mice.

10.1128/mSystems.00070-16.1 Figure S1  Bar graph of the LDA score for bacterial species that are more abundant in vehicle- and Gly-MCA-treated mice (A), mice treated with vehicle versus the Gly-MCA plus GW4064 treatment group (B), and the Gly-MCA-treated mice versus those treated with Gly-MCA plus GW4064 (C). Download Figure S1, TIF file, 1.4 MB. Copyright © 2016 Zhang et al. 2016 Zhang et al. This content is distributed under the terms of the Creative Commons Attribution 4.0 International license.

**FIG 2:**
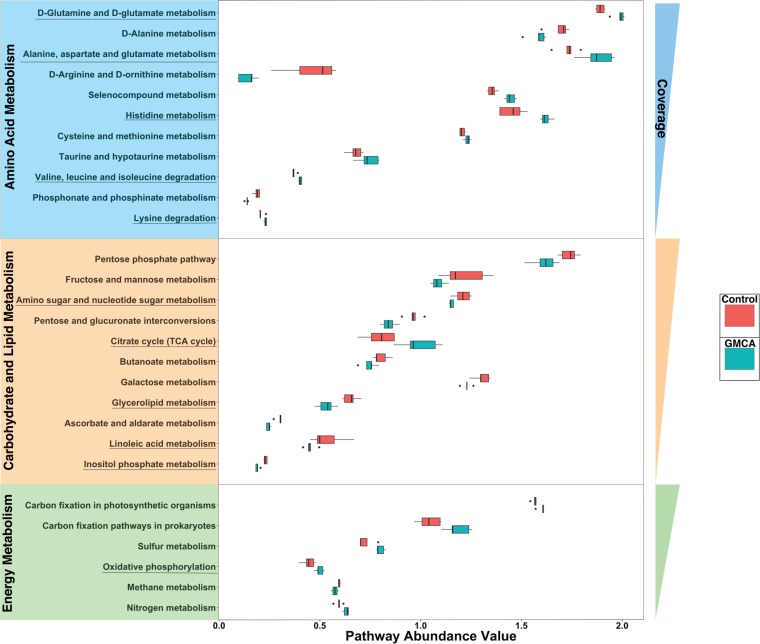
PICRUSt analysis results of predicted functional pathways in the gut microbiota. Pathways are grouped based on the following categories: amino acid metabolism (blue), carbohydrate and lipid metabolism (yellow), and energy metabolism (green). The pathway abundance values for control (red) and Gly-MCA treatment (dark green) are representative of the amount of genes and normalized to the total number of genes present in a particular pathway from each sample. These pathways were also ordered by decreasing coverage, which was calculated based on the total possible amount of genes (according to the Metacyc database). The underlined predicted functional pathways highlighted were supported by metabolomics analyses of liver extracts. All pathways shown are significant according to LEfSe. LEfSe uses the Kruskal-Wallis test and also the Wilcoxon test at a cutoff of 0.05 to determine significant and biologically relevant pathways between two groups.

**FIG 3:**
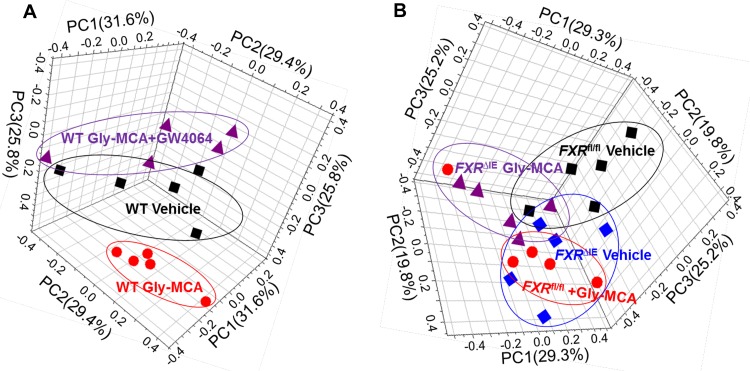
NMR metabolomics analysis results for mouse liver metabolic profiling. (A) Three-dimensional PCA score plot from hepatic metabolomes of vehicle-treated mice, Gly-MCA-treated mice, and Gly-MCA-treated mice administered GW4064. (B) Three-dimensional PCA score plot from hepatic metabolomes of *Fxr*^fl/fl^ and *Fxr*^ΔIE^ mice with and without Gly-MCA treatment.

To predict the abundance of gene families and related functional pathways of microbial communities in the cecal contents, PICRUSt (**p**hylogenetic **i**nvestigation of **c**ommunities by **r**econstruction of **u**nobserved **st**ates), a predictive metabolism approach, was performed based on the 16S rRNA gene sequencing and the Green Genes database (Fig. 2). The results suggested that many bacterial pathways involved in amino acid, carbohydrate, lipid, and energy metabolism were significantly modulated by Gly-MCA treatment. The underlined pathways in Fig. 2 were supported by the subsequent NMR-based metabolomics analyses (Fig. 3; see also Fig. S3 and S4 in the supplemental material).

### Gly-MCA reduces obesity through modulation of gut microbiota composition and intestinal FXR signaling.

Previous studies demonstrated that intestinal FXR modulation by agonist or antagonist resulted in a broad metabolic improvement of NAFLD and obesity (10, 11, 23, 24). In the current study, a ^1^H NMR-based metabolomics approach was used to measure metabolic alterations in the livers of HFD-fed mice treated with Gly-MCA. Intestinal *Fxr*-null (*Fxr*^ΔIE^) mice and control (*Fxr*^fl/fl^) mice on an HFD were used to determine the role of intestinal FXR in the Gly-MCA-improved metabolic phenotype. ^1^H NMR spectra were recorded for liver extracts obtained from the vehicle and Gly-MCA treatment groups (see Fig. S2 in the supplemental material). The resonances were assigned to specific metabolites based on previous data and further confirmed individually by a series of two-dimensional (2D) NMR experiments (see Table S2 in the supplemental material) (25). The scores plot from a principal component analysis (PCA) showed intergroup metabolic differences, where each point represented a mouse liver metabolome and the distance between data points reflected the scale of their metabolic differences (Fig. 3). This experiment revealed that Gly-MCA treatment induced significant metabolic changes in the livers of HFD-fed mice (Fig. 3A), whereas treatment with GW4064 reversed the changes found in the Gly-MCA-treated HFD-fed mouse group (Fig. 3A). Furthermore, both HFD-fed *Fxr*^ΔIE^ mice and Gly-MCA-treated, HFD-fed *Fxr*^fl/fl^ mice exhibited metabolic differences (Fig. 3B) in comparison with *Fxr*^fl/fl^ vehicle-treated mice, and no significant differences were observed in the liver metabolome obtained from *Fxr*^ΔIE^ mice with or without Gly-MCA treatment and that of Gly-MCA-treated *Fxr*^fl/fl^ mice (Fig. 3B).

10.1128/mSystems.00070-16.2 Figure S2  Representative 600-MHz ^1^H NMR spectra for liver aqueous extracts from vehicle (A), Gly-MCA-treated mice (B), *Fxr*^fl/fl^ vehicle (C), and Gly-MCA-treated *Fxr*^fl/fl^ mice (D). The region of δ 5.1 to 9.20 in the liver spectra was vertically expanded 8 times compared with the region of δ 0.6 to 4.4. Keys: 1, lipid; 2, isoleucine; 3, leucine; 4, valine; 5, d-3-hydroxybutyrate; 6, lactate; 7, alanine; 8, acetate; 9, lysine; 10, glutamate; 11, glutamine; 12, glutathione; 13, succinate; 14, pyruvate; 15, aspartate; 16, choline; 17, phosphorylcholine; 18, glycerophosphocholine; 19, TMAO; 20, taurine; 21, glucose and amino acids; 22, triglycerides; 23, α-glucose; β-glucose; 24, glycogen; 25, unsaturated fatty acid; 26, uridine; 27, UDP (UDP); 28, inosine; 29, AMP (AMP); 30, fumarate; 31, tyrosine; 32, histidine; 33, phenylalanine; 34, uracil; 35, xanthine; 36, UMP (UMP); 37, hypoxanthine; 38, nicotinamide; 39, betaine; 40, bile acids; 41, inosine-5′-monophosphate (5′-IMP); 42, formate; 43, adenosine. See also Table S2. Download Figure S2, TIF file, 0.9 MB. Copyright © 2016 Zhang et al. 2016 Zhang et al. This content is distributed under the terms of the Creative Commons Attribution 4.0 International license.

**FIG 4:**
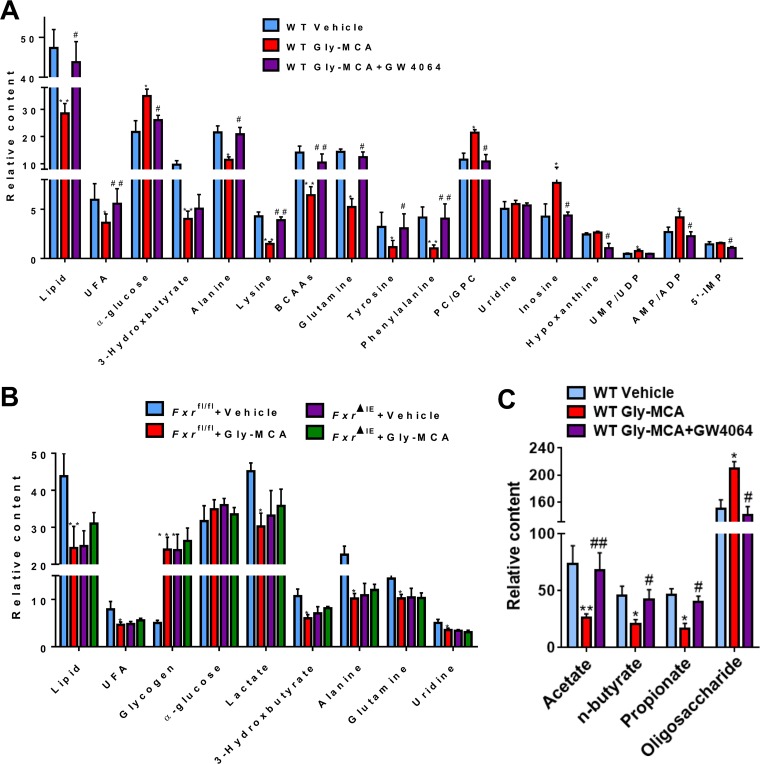
Gly-MCA efficiently ameliorates obesity-related metabolic disorders by FXR inhibition. Relative abundance of the significantly changed metabolites in the liver obtained from vehicle-treated mice, Gly-MCA-treated mice, and Gly-MCA-treated mice administered GW4064 (A); *Fxr*^fl/fl^ and *Fxr*^ΔIE^ mice with and without Gly-MCA treatment (B); SCFAs (acetate, butyrate, and propionate) and oligosaccharides in the cecal contents from vehicle-treated mice, Gly-MCA-treated mice, and Gly-MCA-treated mice administered GW4064 (C). *n* = 5 mice per group. Data are means ± SD (*n* = 5 per group). *, *P* < 0.05; **, *P* < 0.01 (compared with vehicle treatment). #, *P* < 0.05; ##, *P* < 0.01 (compared with Gly-MCA treatment of HFD-fed mice). Data were analyzed via a one-way ANOVA with Tukey’s correction.

Pair-wise comparative orthogonal projection to latent structures with discriminant analysis (OPLS-DA) was conducted to uncover metabolic changes induced by Gly-MCA and the aforementioned treatments (see Fig. S3 and S4 in the supplemental material). Compared with the vehicle-treated control group, Gly-MCA treatment resulted in a significant reduction in the levels of lipids, unsaturated fatty acids (UFA), triglycerides, 3-HB, and several amino acids, including alanine, lysine, glutamine, valine, leucine, isoleucine, tyrosine, and phenylalanine (Fig. 4A; see also Fig. S3A). Gly-MCA treatment also led to significant elevations of glucose, PC/GPC, and some nucleosides and nucleotides, including inosine, UMP, UDP, AMP, and ADP in mouse liver (Fig. 4A; see also Fig. S3A). However, GW4064 treatment reversed these Gly-MCA-induced metabolic changes, especially for lipid, UFA, and PC/GPC, which returned to their initial levels in HFD-fed mice (Fig. 4A; see also Fig. S3B and C). Compared with *Fxr*^fl/fl^ vehicle-treated mice, *Fxr*^ΔIE^ HFD-fed mice with or without Gly-MCA treatment and Gly-MCA-treated *Fxr*^fl/fl^ HFD-fed mice exhibited lower levels of lipids, UFA, lactate, 3-HB, alanine, glutamine, and uridine, but higher levels of glycogen in the liver (Fig. 4B; see also Fig. S4A and B). In particular, no significant differences were observed in the levels of these metabolites obtained from the livers of *Fxr*^ΔIE^ HFD-fed mice treated with or without Gly-MCA versus Gly-MCA-treated *Fxr*^fl/fl^ HFD-fed mice (Fig. 4B; see also Fig. S4C and D).

10.1128/mSystems.00070-16.3 Figure S3  O-PLS-DA scores (left) and correlation coefficient-coded loading plots for the models (right) from NMR data of aqueous liver extracts, discriminating between the Gly-MCA-treated mice and wild-type (WT) mice treated with vehicle (A) or Gly-MCA-treated mice and mice in the Gly-MCA plus GW4064 treatment group (B). Download Figure S3, TIF file, 1.4 MB. Copyright © 2016 Zhang et al. 2016 Zhang et al. This content is distributed under the terms of the Creative Commons Attribution 4.0 International license.

10.1128/mSystems.00070-16.4 Figure S4  O-PLS-DA scores (left) and correlation coefficient-coded loading plots for the models (right) from NMR data of aqueous liver extracts, discriminating between *Fxr*^fl/fl^ vehicle-treated and Gly-MCA-treated *Fxr*^fl/fl^ mice (A), *Fxr*^ΔIE^ vehicle-treated and *Fxr*^fl/fl^ vehicle-treated mice (B), *Fxr*^ΔIE^ vehicle-treated and Gly-MCA-treated *Fxr*^ΔIE^ mice (C), and Gly-MCA-treated *Fxr*^ΔIE^ mice and Gly-MCA-treated *Fxr*^fl/fl^ mice (D). Download Figure S4, TIF file, 1.8 MB. Copyright © 2016 Zhang et al. 2016 Zhang et al. This content is distributed under the terms of the Creative Commons Attribution 4.0 International license.

Compared with the vehicle-treated HFD-fed mice, Gly-MCA treatment significantly decreased the levels of short-chain fatty acids (SCFAs, e.g., acetate, propionate, and *n*-butyrate) coupled with elevated levels of oligosaccharides in the cecal contents (Fig. 4C; see also Fig. S5A in the supplemental material). However, a significant reversal in the levels of SCFAs and oligosaccharides was observed in the cecal contents of HFD-fed mice treated with Gly-MCA plus GW4064 (Fig. 4C; see also Fig. S5B). These results further suggest that Gly-MCA treatment modulates the gut microbiota and its associated fermentation function.

10.1128/mSystems.00070-16.5 Figure S5  O-PLS-DA scores (left) and correlation coefficient-coded loading plots for the models (right) from NMR data of aqueous cecal content extracts discriminating between the Gly-MCA-treated group and wild-type (WT) vehicle-treated mice (A) or the Gly-MCA-treated group versus Gly-MCA plus GW4064-treated group (B). Download Figure S5, TIF file, 0.9 MB. Copyright © 2016 Zhang et al. 2016 Zhang et al. This content is distributed under the terms of the Creative Commons Attribution 4.0 International license.

**FIG 5:**
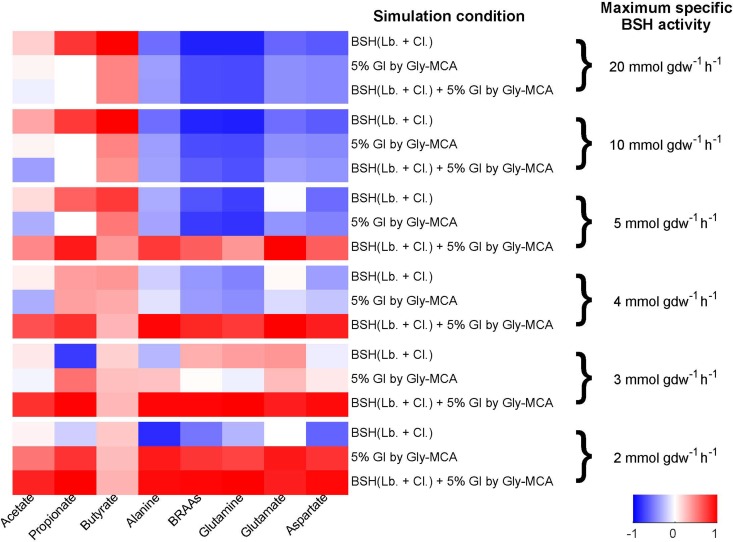
Correlation between metabolite levels and profiles of metabolite consumption and production in the cecal contents, as predicted by genome-scale metabolic modeling tested at different levels of maximum BSH activity. The heat map of the Pearson correlation coefficients between the predicted production or consumption and the experimentally measured levels across all 10 samples is shown for each of the short-chain fatty acids and amino acids. Statistical significance was determined by transforming the Pearson *r* value into the *t* value, and then the t distribution was used to find the *P* value. Correlation values above 0.63 are statistically significant (*P* < 0.05).

To further explore the effect of Gly-MCA on the gut microbiota, a community genome-scale metabolic model comprised of 10 representative species (see Table S3 in the supplemental material) was compiled to analyze metabolites generated by the gut microbiota (Fig. 5). Bile salt-hydrolyzing pathways were added to the metabolic models of those representative organisms in the *Clostridia* class and *Lactobacillus* species to account for any potential effects of Gly-MCA on BSH activity and the microbiome (19). The community model was constrained by the ratios between the species abundances from the 16S rRNA gene sequencing data for each sample (Fig. 5). Two working hypotheses were incorporated into the model: (i) the total BSH activity was assumed to be proportional to the abundances in the community of *Clostridia* and *Lactobacillus*; (ii) the growth of the gut microbial community treated with Gly-MCA was inhibited by 5%, based on experimental observations. In untreated samples, simulated maximum growth of the gut microbiota requires the production of SCFAs and consumption of amino acids, supporting the notion that amino acids act as the substrates rather than products of the gut microbiota. In the treated samples, the required SCFA production and amino acid consumption decreased. From the simulation results repeated with various levels of maximum BSH activity (Fig. 5), as long as the BSH activity was not in excess in all samples (i.e., the gut microbiota cannot consume high levels of bile salts regardless of the *Clostridia* and *Lactobacillus* abundances), the predicted change in the metabolite production and consumption showed a significant positive correlation (*r* > 0.63) with the measured levels of SCFAs and amino acids in the cecum. The exception to this correlation was butyrate, which was predicted to have particularly high production in one of the untreated samples. The change in the simulated metabolite production and consumption in the presence of Gly-MCA can be attributed to the two model assumptions. First, when the overall growth in the presence of Gly-MCA is inhibited by 5%, the consumption of SCFAs and the production of amino acids that are coupled to microbial growth decrease, as expected when the biomass level is decreased. Second, the decrease in BSH activity upon Gly-MCA treatment as a result of the decrease in the abundances of *Clostridia* and *Lactobacillus* plays a role in the metabolite abundances observed. The taurine released from taurine-conjugated bile salts provides extra carbon and nitrogen that can support additional microbial growth. Sulfite, a product of taurine degradation, can act as an electron receptor for the gut microbiota. Without the assumption that BSH activity is proportional to the abundance of *Clostridia* and *Lactobacillus*, the model predicts a higher biomass yield of the gut microbiota in mice on an HFD treated with Gly-MCA compared to that in the untreated group. The decreased BSH activity limits the microbial growth in the treated samples, therefore limiting those SCFA production and amino acid consumption levels coupled to microbial growth (Fig. 5). The modeling analysis supports the hypothesis that Gly-MCA inhibits the overall microbial fermentation and in particular the BSH activity associated with *Clostridia* and *Lactobacillus*.

**FIG 6:**
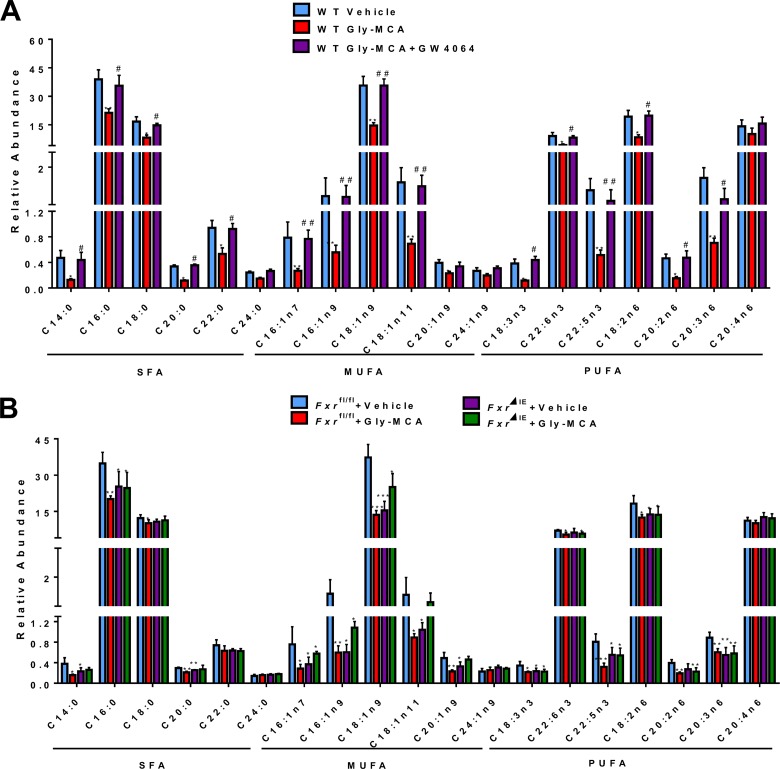
Gly-MCA efficiently improves fatty acid metabolism in HFD-induced obesity by FXR inhibition. Fatty acid composition in the liver of vehicle-treated mice, Gly-MCA-treated mice, and Gly-MCA-treated mice administered GW4064 treatment (A) and in *Fxr*^fl/fl^ and *Fxr*^ΔIE^ mice with and without Gly-MCA treatment (B). Fatty acids were extracted by the Folch method and quantified by GC-MS. Data are means ± SD (*n* = 5 per group). *, *P* < 0.05; **, *P* < 0.01 (compared with vehicle treatment). #, *P* < 0.05; ##, *P* < 0.01 (compared with Gly-MCA treatment of HFD-fed mice). Data were analyzed with a one-way ANOVA with Tukey’s correction.

Long-chain fatty acids (LCFAs) are associated with the development of NAFLD and hepatic steatosis. Here, total fatty acid composition was analyzed by gas chromatography-mass spectrometry (GC-MS), which revealed that Gly-MCA treatment led to significant decreases in the levels of total fatty acids, including saturated fatty acids (SFA), UFA, and polyunsaturated fatty acids (PUFA); GW4064 reversed these changes in fatty acid levels (Fig. 6A) in the livers of HFD-fed mice. Furthermore, Gly-MCA treatment also resulted in a significant reduction in LCFAs in the livers of *Fxr*^fl/fl^ HFD-fed mice, whereas the levels of LCFAs remained similar in the livers of *Fxr*^fl/fl^ and *Fxr*^ΔIE^ HFD-fed mice upon Gly-MCA treatment and also in vehicle-treated HFD-fed *Fxr*^ΔIE^ mice (Fig. 6B).

To further investigate the mechanism by which Gly-MCA ameliorates obesity-related metabolic disorders, the expression levels of mRNAs encoding enzymes involved in lipid, bile acids, and glucose metabolism were determined. Gly-MCA treatment resulted in significant downregulation in the expression of lipid and fatty acid synthesis-related mRNAs, including sterol response element-binding protein 1c (*Srebp1c*), DNA fragmentation factor α-like effector A (*Cidea*), fatty acid synthase (*Fasn*), and acetyl coenzyme A (CoA) carboxylase 1 (*Acaca*), elongation of very-long-chain fatty acids protein 5 (*Elovl5*), and elongation of very-long-chain fatty acids protein 6 (*Elovl6*) in the livers of HFD-fed mice (see Fig. S6A and B in the supplemental material). A significant decrease in mRNAs encoding the triglyceride and cholesterol biosynthetic enzymes, including diacylglycerol *O*-acyltransferase 1 (*Dgat1*), diacylglycerol *O*-acyltransferase 2 (*Dgat2*), 3-hydroxy-3-methylglutaryl-CoA reductase (*Hmgcr*), and 3-hydroxy-3-methylglutaryl-CoA synthase 1 (*Hmgcs1*) was also found in the livers of Gly-MCA-treated HFD-fed mice (see Fig. S6B). Furthermore, the expression of mRNAs encoded by genes, such as *Cyp7a1*, *Cyp7b1*, and *Cyp27a1*, involved in bile acid synthesis was significantly increased by Gly-MCA treatment (see Fig. S6C). Consistently, our previous measurements of bile acid composition revealed that Gly-MCA upregulated T-α-MCA and T-β-MCA levels in the ileum and liver of HFD-fed mice (11). Interestingly, GW4064 treatment suppressed expression of all the mRNAs that were increased after Gly-MCA treatment (see Fig. S6A to C). Similarly, Gly-MCA treatment decreased the levels of mRNAs encoding lipid, fatty acid, triglyceride, and cholesterol synthesis, whereas increased bile acid synthesis-related mRNAs were found in the livers of HFD-fed *Fxr*^fl/fl^ mice (see Fig. S6D to F). Compared with HFD-fed *Fxr*^fl/fl^ mice, HFD-fed *Fxr*^ΔIE^ mice expressed significantly lower levels of mRNAs encoded by genes involved in lipid, fatty acid, triglyceride, and cholesterol synthesis but higher levels of bile acid synthesis-related mRNAs (see Fig. S6D to F). However, no significant differences in the expression profiles of these genes in the liver (see Fig. S6D to F) were observed between Gly-MCA-treated HFD-fed *Fxr*^ΔIE^ mice and vehicle-treated HFD-fed *Fxr*^ΔIE^ mice. In addition, HFD-fed *Fxr*^ΔIE^ mice exhibited significantly lower levels of *Srebp1* and *Cidea*, which are involved in lipid synthesis and inflammatory factors such as LCN2, interleukin-1β, tumor necrosis factor alpha, and Saa1 in the adipose tissue in comparison with levels in HFD-fed *Fxr*^fl/fl^ mice (see Fig. S7 in the supplemental material). However, no significant differences in the expression profiles of these genes in the adipose tissues were observed between Gly-MCA-treated HFD-fed *Fxr*^ΔIE^ mice and vehicle-treated HFD-fed *Fxr*^ΔIE^ mice. These data demonstrated that the improvements of obesity-related metabolic disorders by Gly-MCA treatment are mainly due to inhibition of intestinal FXR signaling.

10.1128/mSystems.00070-16.6 Figure S6  Gly-MCA reduces mRNAs levels related to lipid, fatty acid, triglyceride, and bile acid metabolism in HFD-induced obesity through inhibition of FXR activity. Analysis of mRNA levels of *Srebp1c*, *Cidea*, *Acaca*, *Fasn*, *Elovl5*, and *Elovl6* under conditions of chemical agonism/antagonism in wild type mice (A) or agonism in wild-type or in *Fxr*^ΔIE^ mice (B). Analysis of mRNA levels of *Dgat1*, *Dgat2*, *Hmgcr*, and *Hmgcs1* are shown in panels C and F. Analysis of mRNA levels of *Cyp7a1*, *Cyp7b1*, *Cyp8b1*, and *Cyp27a1* in the liver of vehicle-treated mice, Gly-MCA-treated mice, and Gly-MCA-treated mice administered GW4064 (A, B, and C) or in *Fxr*^fl/fl^ and *Fxr*^ΔIE^ mice with or without Gly-MCA treatment (D, E, and F). Data are presented as means ± SD (*n* = 5 per group). *, *P* < 0.05; **, *P* < 0.01 (compared with vehicle treatment). #, *P* < 0.05; ##, *P* < 0.01 (compared with Gly-MCA treatment of HFD-fed mice). A one-way ANOVA with Tukey’s correction was used. Download Figure S6, TIF file, 1.4 MB. Copyright © 2016 Zhang et al. 2016 Zhang et al. This content is distributed under the terms of the Creative Commons Attribution 4.0 International license.

10.1128/mSystems.00070-16.7 Figure S7  Gly-MCA reduces mRNAs levels related to lipid metabolism and inflammation in HFD-induced obesity through inhibition of FXR activity. Shown are results of the analysis of mRNA levels of *Srebp1c*, *Cidea*, *Lcn2*, *IL-1β*, *Tnf-α*, and *Saa1* in adipose tissue of HFD-fed *Fxr*^fl/fl^ mice with and without Gly-MCA treatment and HFD-fed *Fxr*^ΔIE^ mice with and without Gly-MCA treatment. Data are means ± SD (*n* = 5 per group); *, *P* < 0.05; **, *P* < 0.01 (compared with vehicle treatment). #, *P* < 0.05; ##, *P* < 0.01 (compared with Gly-MCA treatment of HFD-fed mice). Data were analyzed using a one-way ANOVA with Tukey’s correction. Download Figure S7, TIF file, 0.9 MB. Copyright © 2016 Zhang et al. 2016 Zhang et al. This content is distributed under the terms of the Creative Commons Attribution 4.0 International license.

### Relationship between the gut microbiome and host metabolome after Gly-MCA treatment.

**FIG 7:**
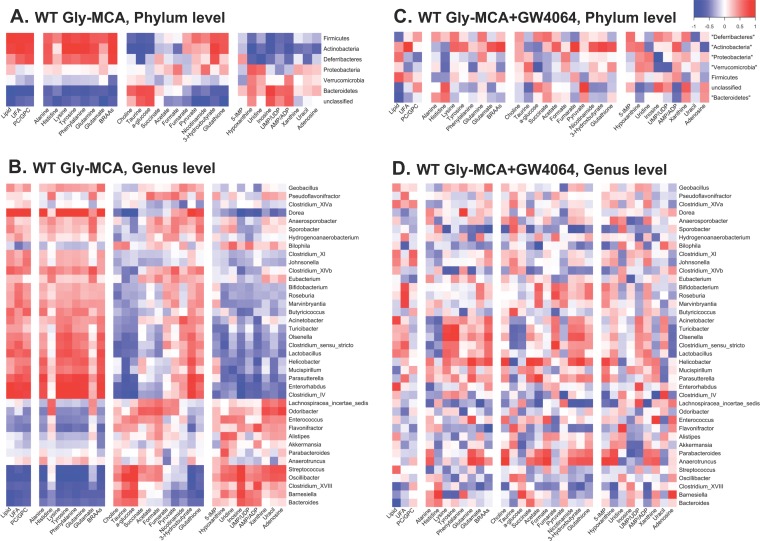
Relationship between gut microbiome and host metabolome. A Pearson correlation analysis was used to investigate the relationships between bacterial populations and metabolite levels after Gly-MCA treatment (with and without GW4064). Statistical significance was determined by transforming the Pearson *r* value into the *t* value and then using the *t* distribution to find the *P* value. Correlation values above 0.63 or below −0.63 were statistically significant. Heat maps of the correlation between the gut microbiota and metabolites from Gly-MCA-treated mice (A and B) and Gly-MCA-treated and then GW4064-treated mice (C and D). Results are shown for phyla (A and C) and genera (B and D).

To explore the functional correlation between the gut microbiome changes and host metabolome alterations, a correlation matrix was generated by calculating Pearson’s correlation coefficients. Clear correlations could be identified between modulated gut microbiomes and altered metabolic profiles (*r* > 0.63 or < −0.63). The resulting association maps indicated positive and negative correlations between the levels of host liver metabolites and the gut microbiomes of Gly-MCA-treated mice in comparison with vehicle-treated mice (Fig. 7A and B). Of particular note, some metabolites, including lipid, UFA, PC/GPC, alanine, histidine, lysine, tyrosine, phenylalanine, glutamine, glutamate, branched amino acids, pyruvate, nicotinamide, 3-HB, and glutathione, which decreased in the livers of HFD-fed mice after Gly-MCA treatment, were negatively correlated with the presence of phylum *Bacteroidetes* (Fig. 7A) and genera *Streptococcus*, *Oscillibacter*, *Bacteroides*, *Clostridium* cluster XVIII, and *Barnesiella* (Fig. 7B) but positively correlated with the phyla *Firmicutes*, *Actinobacteria*, and *Deferribacteres* (Fig. 7A) and the genera *Lactobacillus*, *Helicobacter*, *Enterorhabdus*, and *Clostridium* IV (Fig. 7B). Furthermore, other metabolites, including choline, taurine, glucose, and some nucleosides, which increased in the livers of Gly-MCA-treated HFD-fed mice, were negatively correlated with the phyla *Firmicutes* and *Actinobacteria* and at the genus level for *Lactobacillus*, *Enterorhabdus*, and *Clostridium* IV but were positively correlated with the phylum *Bacteroidetes* and genera *Streptococcus*, *Oscillibacter*, *Bacteroides*, *Clostridium* XVIII, and *Barnesiella* (Fig. 7A and B). However, no significant correlation was observed between the gut microbiota and hepatic metabolome in the Gly-MCA-treated HFD-fed mice after GW4064 administration (Fig. 7C and D). These observations indicated that the significantly modulated gut microbiota after Gly-MCA treatment correlated with the improvement of obesity-related metabolic disorders in the liver.

## DISCUSSION

Gly-MCA, a potent FXR antagonist, improves obesity, insulin resistance, and steatosis (11). However, the underlying mechanisms and the role of the gut microbiota in the metabolic effects of Gly-MCA remain undetermined. In the current study, modulation of the gut microbiota by Gly-MCA and changes in cometabolites of host and gut microbiota were observed. At the phylum level, Gly-MCA-treated mice fed an HFD exhibited lower proportions of *Firmicutes* and *Actinobacteria* but a higher proportion of *Bacteroidetes* than observed in vehicle-treated HFD-fed mice, indicating that the total population of the gut microbiota was modulated following Gly-MCA treatment. More recent evidence suggests that an increased ratio of *Firmicutes* to *Bacteroidetes* may trigger primary contributions to the pathogenesis of obesity (26). In a murine model, it was shown that the relative abundance of these two predominant members, *Firmicutes* and *Bacteroidetes*, differs among lean and obese mice; the obese mouse has a higher proportion of *Firmicutes* to *Bacteroidetes* (50% greater) than the lean mouse (27). Previous studies in humans also revealed a lower proportion of *Bacteroidetes* and higher proportion of *Actinobacteria* in obese versus lean Europeans or individuals of African ancestry (28, 29). A previous study of *Fxr*-null mice (30) revealed that the relative abundances of *Bacteroidetes* and *Firmicutes* were similar to what was observed with Gly-MCA treatment in the current study. Here significant depletion of the class *Clostridia*, family *Lactobacillaceae* and genus *Lactobacillus* was observed in HFD-fed mice after Gly-MCA treatment, which is consistent with previous studies showing that *Lactobacillus* populations are elevated in obese mice and decrease after gastric bypass surgery (31, 32). Furthermore, members of the class *Clostridia*, family *Lactobacillaceae*, and genus *Lactobacillus* were associated with BSH enzymatic activity, deconjugation of taurine-conjugated bile acids, such as T-β-MCA, and the development of obesity (19). A previous study reported that treatment of HFD-fed mice with antibiotics or the antioxidant tempol resulted in a significant reduction of the genus *Lactobacillus* and its BSH activity, coincident with accumulation of the endogenous FXR antagonist T-β-MCA (9, 10). Microbial metabolism of T-β-MCA to β-MCA by BSH is associated with obesity, insulin resistance, and fatty acid disease (9, 18). Importantly, T-β-MCA and β-MCA do not exist in humans; thus, there is limited translational potential for T-β-MCA in humans. Accordingly, Gly-MCA, a new high-affinity intestinal FXR antagonist, was designed that is structurally and functionally similar to T-β-MCA and demonstrated stability in the gut and resistance to hydrolysis into β-MCA by BSH and improvement of HFD-induced obesity and insulin resistance (11). Furthermore, clinical studies showed that ursodeoxycholic acid (UDCA), another FXR antagonist, has already been used for NAFLD therapy (14, 15). These findings suggest that Gly-MCA might similarly have translational potential for human obesity and NAFLD conditions.

Here, HFD-fed *Fxr*^ΔIE^ mice exhibited a similar metabolic phenotype as both *Fxr*^fl/fl^ and *Fxr*^ΔIE^ HFD-fed mice treated with Gly-MCA. Furthermore, administration of a synthetic high-affinity FXR agonist, GW4064, reversed the metabolic changes in livers of Gly-MCA-treated mice fed an HFD. These observations indicated that the gut microbiota contributing to the improvement of obesity by Gly-MCA requires intestinal FXR signaling, which plays a central role in Gly-MCA efficacy. Consistently, a recent study revealed that the altered gut microbiota in HFD-fed *Fxr*-deficient mice may directly contribute to the obese phenotype (30). However, these data cannot exclude the possibility that GW4064 treatment alone alters the gut microbiota population as a result of modulation of hepatic FXR signaling.

Normally, HFD-induced obesity arises when energy intake, principally stored as lipids, fatty acids, and triglycerides, exceeds energy expenditure (6). Here Gly-MCA treatment induced a significant reduction of hepatic lipid and fatty acid levels, suggesting efficient suppression of lipid and fatty acid synthesis in HFD-fed mice, which was confirmed by the downregulation in expression of lipid and fatty acid synthesis-related genes, including *Srebp1c*, *Cidea*, *Fasn*, *Acaca*, *Elovl5*, and *Elovl6*. Further, hepatic triglyceride and cholesterol concentrations are another metabolic variable of obesity and have been linked to large amounts of SCFAs, such as acetate, butyrate, and propionate, providing an additional source of energy for the body (33). SCFAs are end products of bacterial fermentation and are known to stimulate triglyceride and cholesterol synthesis in the liver via binding to G-coupling proteins, such as GPCR41 and GPCR43 (34, 35). In the current study, a significant depletion of SCFAs coupled with elevation of glucose and oligosaccharides in the cecal contents of HFD-fed mice after Gly-MCA treatment suggested suppression of bacterial fermentation by Gly-MCA, thus inhibiting hepatic triglyceride and cholesterol *de novo* biosynthesis. Interestingly, a previous study revealed that the genus *Lactobacillus* is positively correlated with hepatic triglyceride biosynthesis (36), which is in agreement with the present results that Gly-MCA decreased the family *Lactobacillaceae* and genus *Lactobacillus* in the cecal contents and hepatic triglyceride levels. Genome-scale metabolic modeling of the gut microbiota further supported and rationalized the observed changes in metabolites and suppression of bacterial fermentation in cecal contents caused by Gly-MCA. Decreasing the BSH activity proportionally with the abundances of *Lactobacillus* and *Clostridia* decreases the SCFAs consumed and amino acids produced, mimicking the changes in metabolite levels in cecal contents. The consistency between modeling predictions and experimental observations led to new hypotheses regarding the mechanism of Gly-MCA action and the effects of various levels of BSH activity on the extent of suppressed bacterial fermentation.

Emerging findings in literature suggest a cross talk between the gut microbiota and liver (gut-liver axis) that impacts the development of metabolic syndrome, including obesity and fatty liver diseases (37). In this study, an integrative analysis demonstrated a strong correlation between gut microbiota and obesity-related metabolic pathways, such as lipid, glucose, and amino acid metabolism in the liver. For example, hepatic lipid, fatty acids, and amino acids were negatively correlated with members of the phylum *Bacteroidetes* and positively correlated with members of the phyla *Firmicutes* and *Actinobacteria* in the cecal contents of HFD-fed mice after Gly-MCA treatment. In particular, the genus *Lactobacillus* positively correlated with the levels of hepatic lipids, fatty acids, and amino acids, indicating that the genus *Lactobacillus* may have a vital role in host energy metabolism. Results from the present study demonstrate that the gut microbiota-mediated pathways, including amino acid metabolism, carbohydrate and lipid metabolism, and energy metabolism, are significantly modulated by Gly-MCA treatment. Specifically, some amino acids, including glutamine, glutamate, histidine, lysine, and branched-chain amino acids (BCAAs) metabolism and their degradation were all increased in the guts of mice treated with Gly-MCA. This result is most likely explained by the fact that these amino acids are decreased in the livers of Gly-MCA-treated mice, as verified by ^1^H NMR metabolomics analyses. Of particular note, Gly-MCA regulated energy homeostasis of HFD-diet fed mice and therefore amino acids, including alanine, lysine, BCAAs, glutamine, tyrosine, and phenylalanine, major energy sources for the whole body, were significantly affected by Gly-MCA treatment and moreso than others. Interestingly, almost all measured amino acids are negatively correlated with *Bacteroidetes*, which significantly increased in the mice treated with Gly-MCA. The increased levels of amino acid metabolism in the gut could be due at least in part to the increased levels of *Bacteroidetes* species in the cecal content. Similarly, the predicted pathways, such as glucose, the tricarboxylic acid (TCA) cycle, lipid, and energy metabolism in the gut were also significantly modulated by Gly-MCA and strongly supported by metabolomics results in the liver. These observations reveal the underlying mechanism by which inhibition of intestinal FXR signaling by Gly-MCA modulates the gut microbiota and improves host lipid metabolism, thus reducing diet-induced obesity and fatty liver disease. In future studies, extension of genome-scale metabolic modeling to include the host liver metabolism in modeling the exchange between host and microbial metabolites will be a promising way to predict and understand the observed correlation between the gut microbiota and host metabolism, e.g., *Lactobacillus* and the levels of hepatic triglycerides and *Bacteroidetes* and the levels of hepatic amino acids.

In conclusion, the present study revealed that modulation of the gut microbiota by Gly-MCA improves diet-induced obesity and associated phenotypes through effects on the host lipid metabolic profile. Notably, these altered obesity-related metabolic pathways were found to be highly associated with intestine-specific FXR signaling. These findings demonstrated that Gly-MCA has beneficial effects on obesity through the modulation of the gut microbiota and intestinal FXR signaling and could be developed as a new drug to treat fatty liver disease.

## MATERIALS AND METHODS

### Animal studies.

All animal studies were performed in accordance with the Institute of Laboratory Animal Resources guidelines and reviewed and approved by the NCI Animal Care and Use Committee. Mice were treated humanely and with regard for the alleviation of suffering. Male C57BL/6N mice (25 g body weight; 6 weeks old) were obtained from the Mouse Repository (NCI, Frederick, MD). Littermate intestine-specific *Fxr*-null (*Fxr*^ΔIE^) mice and control (*Fxr*^fl/fl^) mice were developed on a C57BL/6N genetic background (over 10 generations). Mice were housed individually in their home cages in temperature- and light-controlled rooms and given water and food *ad libitum*. All mice in this study were housed in the same room of the same vivarium in order to avoid differences in gut microbiota. Dough pills containing Gly-MCA synthesized as described previously (11) were prepared with tablet molds, and one pill uniformly contained 0.25 mg Gly-MCA, thus providing a final dose of 10 mg/kg of body weight for a mouse. The mice were trained to eat the dough pills prior to the study. A total of 15 mice fed an HFD (60% kcal from fat; Bio-Serv, Inc.) were divided into 3 groups: vehicle, Gly-MCA, and GW4064 plus Gly-MCA (for which each pill contained 0.25 mg GW4064 and 0.25 mg Gly-MCA) and treated for 5 weeks. Similarly, a total of 20 male *Fxr*^fl/fl^ and *Fxr*^ΔIE^ mice fed an HFD were also divided into 4 groups with and without Gly-MCA treatment for 8 weeks, respectively. Liver, intestine, and cecal content samples were collected immediately following CO_2_ asphyxiation and stored at −80°C until analysis.

### RNA isolation and quantitative real-time PCR.

RNA was extracted from frozen liver tissues (~50 mg) using TRIzol reagent (Invitrogen). cDNA was synthesized from 1 µg of total RNA using qScript cDNA SuperMix (Quanta Biosciences), and the products were diluted to 1:10 before use in subsequent reactions. Gene-specific primers were used in each reaction mixture, and all results were normalized to the ribosomal protein β-actin mRNA (primer sequences can be found in Table S1 in the supplemental material). Quantitative PCR (QPCR) assays were carried out using SYBR green QPCR master mix with an ABI Prism 7900HT Fast real-time PCR sequence detection system (Applied Biosystems). The reactions products were analyzed with the ΔΔ*C_T_* method.

10.1128/mSystems.00070-16.8 Table S1  Primer sequences for qRT-PCR. Download Table S1, DOCX file, 0.02 MB. Copyright © 2016 Zhang et al. 2016 Zhang et al. This content is distributed under the terms of the Creative Commons Attribution 4.0 International license.

10.1128/mSystems.00070-16.9 Table S2  ^1^H NMR chemical shifts for metabolites assigned in liver extracts. Download Table S2, DOCX file, 0.03 MB. Copyright © 2016 Zhang et al. 2016 Zhang et al. This content is distributed under the terms of the Creative Commons Attribution 4.0 International license.

10.1128/mSystems.00070-16.10 Table S3  The 10 representative species used in the community metabolic model of the gut microbiome (consisting of 10 representative species with published genome-scale reconstructions). Download Table S3, DOCX file, 0.02 MB. Copyright © 2016 Zhang et al. 2016 Zhang et al. This content is distributed under the terms of the Creative Commons Attribution 4.0 International license.

### Gut microbiota analysis.

The bacteria in the cecal contents were extracted using the EZNA stool DNA kit (OMEGA bio-tek) according to the manufacturer’s instructions. All extracted DNA samples were kept at −20°C until further analysis. PCR amplification was performed on the bacterial genomic DNA samples by using the V4V4 primer set. PCR mixtures were initially heated to 94°C for 3 min, followed by 20 cycles of 94°C for 15 s, 55°C for 45 s, and 72°C for 60 s. Reactions were completed at 72°C for 8 min. The PCR products (~350 bp) were run on a 1% agarose gel to check amplification. PCR products were sent to the Penn State Genomics Core Facility (University Park, PA) for library preparation. Sequencing was performed on an Illumina Miseq system. 16S rRNA gene sequencing analysis was performed using the mothur platform (43) and aligned with the Green Genes and SILVA databases. A biom file was created (using the Green Genes database) and then uploaded onto the Huttenhower galaxy page as described previously (38). PICRUSt analysis was done on the biom file (39). The resulting biom file was then split and analyzed with humann2 software (40). The resulting abundance files were combined and ordered based on pathway description and coverage, in order to produce a summary of pathway abundance values for each sample.

### ^1^H NMR-based metabolomics experiments.

Sodium chloride, methanol, chloroform, K_2_HPO_4_, and NaH_2_PO_4_ (all analytical grade) were obtained from Sigma-Aldrich Chemical Co. Ltd. (St. Louis, MO). Phosphate buffer (0.1 M K_2_HPO_4_ and NaH_2_PO_4_, pH 7.4) was prepared with K_2_HPO_4_ and NaH_2_PO_4_ for their good solubility and low-temperature stability. Sodium 3-trimethylsilyl[2,2,3,3-d4] propionate (TSP-d4) and D2O (99.9% in D) were purchased from Cambridge Isotope Laboratories (Miami, FL).

Liver tissues (~50 mg) were extracted three times with 600 µl of a precooled methanol-water mixture (2/1, vol/vol) using the PreCellys tissue homogenizer (Bertin Technologies, Rockville, MD). After centrifugation at 11,180 × *g* for 10 min at 4°C, the combined supernatants were dried in a vacuum. Each of the aqueous extracts was separately reconstituted into 600 µl phosphate buffer (K_2_HPO_4_/NaH_2_PO_4_, pH 7.4; 0.1 M; 50% [vol/vol] D_2_O) containing 0.005% TSP-d4 as the chemical shift reference. Following centrifugation, 550 µl of each extract was transferred into a 5-mm NMR tube for NMR analysis. The cecal content samples were directly extracted three times with phosphate buffer. Briefly, samples (~50 mg) were mixed with 600 µl precooled phosphate buffer, vortexed for 30 s, and subjected to three consecutive freeze-thaws, followed by homogenization using the Precellys tissue homogenizer. After centrifugation (11,180 × *g*, 4°C) for 10 min, the supernatants (550 µl) were transferred into 5-mm NMR tubes for NMR analysis.

^1^H NMR spectra of liver and cecal content extracts were acquired at 298 K on a Bruker Avance III 600-MHz spectrometer (operating at 600.08 MHz for ^1^H and at 150.93 MHz for ^13^C) equipped with a Bruker inverse cryogenic probe (Bruker Biospin, Germany). A typical one-dimensional NMR spectrum was acquired for each of all samples, employing the first increment of the NOESY pulse sequence (NOESYPR1D). The 90° pulse was adjusted to 10 µs for each sample, and the water signal was suppressed with a weak continuous-wave irradiation. The 32 K data points were collected for each spectrum with a spectral width of 20 ppm and recycle delay of 2 s. For the purposes of NMR signal assignments, a range of 2D NMR spectra was acquired and processed for selected samples, including ^1^H-^1^H correlation spectroscopy (COSY), ^1^H-^1^H total correlation spectroscopy (TOCSY), ^1^H-^13^C heteronuclear single quantum correlation (HSQC), and ^1^H-^13^C heteronuclear multiple-bond correlation spectra (HMBC).

All free induction decay (FID) rates were multiplied by an exponential function with a 1-Hz line broadening factor prior to Fourier transformation. The spectra were calibrated to TSP-d4 at δ 0.00. After manual phase and baseline corrections, each ^1^H NMR spectrum (δ 0.5 to 9.5) was segmented into bins with an equal width of 0.004 ppm (2.4 Hz) by using the AMIX software package (v3.8; Bruker Biospin, Germany). Region δ 4.60 to 5.15 was discarded for imperfect water saturation. Each bucketed region was then normalized to the total sum of the spectral integrals to compensate for the overall concentration differences prior to statistical data analysis.

Multivariate data analysis was carried out with SIMCAP+ software (version 13.0; Umetrics, Sweden). Briefly, PCA and OPLS-DA were conducted on the normalized NMR data. The OPLS-DA models were validated using a 7-fold cross-validation method, and the quality of the model was described by the parameters R2X and Q2 values (see Fig. S3 to 5 in the supplemental material). After back-transformation of the loadings generated from the OPLS-DA, color-coded correlation coefficient loading plots (MatLab; MathWorks Inc., Natick, MA) were employed to indicate the significance of the metabolite contribution to the class separation, with a “hot” color (e.g., red) being more significant than a “cold” color (e.g., blue). In this study, a cutoff value of |r| > 0.707 (*r* > 0.707 and *r* < −0.707) was chosen for the correlation coefficient for significance based on the discrimination significance (*P* ≤ 0.05).

### Correlation analysis of gut microbiome and host metabolome.

A Pearson correlation analysis was used to investigate the relationships between bacterial populations and metabolite levels after Gly-MCA treatment (with and without GW4064). Statistical significance was determined by transforming the Pearson *r* values into *t* values and then using *t* distributions to determine *P* values. The equation used to find the statistical significant cutoff was $r=t/\sqrt{\left(t^{2}+n-2\right)}$, where *r* is the correlation value and *n* is the number of subjects. In this experiment, *n* was equal to 10. The *t* value was found by using the Excel function tinv (0.05, 8), where 0.05 represents a *P* value of 0.05 and 8 is the degrees of freedom for this experiment.

### GC-MS analysis of total fatty acid composition.

The procedures of sample preparation and fatty acid compositional measurements were carried out as described previously (25). In brief, liver tissues (~50 mg) were mixed with 1 ml of a methanol-chloroform mixture (2/1, vol/vol) with addition of 5 µl internal standards (50 µM C_15:0_ free fatty acid and the methyl ester of C_17:0_) and then homogenized using the Precellys tissue homogenizer (Bertin Technologies, Rockville, MD). After centrifugation (20,187 × *g*, 4°C) for 15 min, the supernatant was collected. A 500-µl volume of saline (0.9%) was added into liver extracts. After vortexing for 5 min and centrifugation (20,187 × *g*, 4°C) for 15 min, the organic layer was transferred into 10-ml glass tube and dried with a brief nitrogen gas flush. After adding 1 ml methanol/HCl (41.5 ml/9.7 ml) and vortexing for 5 min, the solution was incubated overnight at 60°C. The resultant mixture was combined with 5 ml hexane and 5 ml saline. Following vortexing for 5 min, the top layer was collected and dried with nitrogen gas. The resultant residues were redissolved in 200 µl hexane and then transferred to an autosampler vial for GC-MS analysis. Fatty acid metabolites were measured on an Agilent 7890A-5975C GC-MS system (Agilent Technologies, Santa Clara, CA). An HP-5MS (Agilent Technologies) capillary column (30 m, 0.25-mm inner diameter, 0.25-µm film thickness) was employed with helium as a carrier gas at a flow rate of 1 ml/min. Sample injection volume was 0.5 µl with a pressure pulsed split ratio (1:10 split, 10 lb/in^2^). The injection port and detector temperatures were 230°C and 250°C, respectively. The initial column temperature was 80°C, where it was held for 1 min, then increased to 205°C at a rate of 20°C/min, then increased to 220°C at a rate of 2°C/min, and then increased to 310°C at a rate of 15°C/min, where it was held for 2 min. Fatty acids were quantified by comparing integrated peak areas following normalization to the internal standards.

### Statistical data analysis.

All the experimental values are presented as means ± standard deviations (SD). Graphical illustrations and statistical analyses were performed with GraphPad Prism version 6.0. Multiple-group comparisons were performed via a one-way analysis of variance (ANOVA) with Tukey’s correction, and *P* values of <0.05 were considered significant.

### Genome-scale metabolic modeling.

The profile of metabolite consumption and production by the gut microbiome when subjected to a HFD was simulated for each wild-type sample treated with and without Gly-MCA by the genome-scale metabolic models of 10 representative organisms spanning four phyla: *Bacteroidetes*, *Firmicutes*, *Actinobacteria*, and *Proteobacteria* (see Table S3 in the supplemental material). For each sample, community growth was modeled as the maximization of the defined metagenomics-based ratio of the representative organism’s biomass, using flux balance analysis (FBA) (41). Flux variability analysis (FVA) was performed to compute the minimum production of short-chain fatty acids and minimum consumption of amino acids by the community of organisms, with the community biomass fixed at 100% or 95% of its maximum value (42). The Pearson correlation coefficient between the predicted production or consumption and the experimentally measured levels across all 10 samples was calculated for each of the short-chain fatty acids and amino acids.

### Accession number(s).

All data have been deposited in NCBI’s Sequence Read Archive under the accession number PRJNA342660.
