# Supplementary figures and images for: Farnesoid X Receptor Signaling Shapes the Gut Microbiota and Controls Hepatic Lipid Metabolism
Source: mSystems. 2016 Oct 11;1(5):e00070-16. doi: 10.1128/mSystems.00070-16 (PMC5080402; doi:10.1128/mSystems.00070-16)

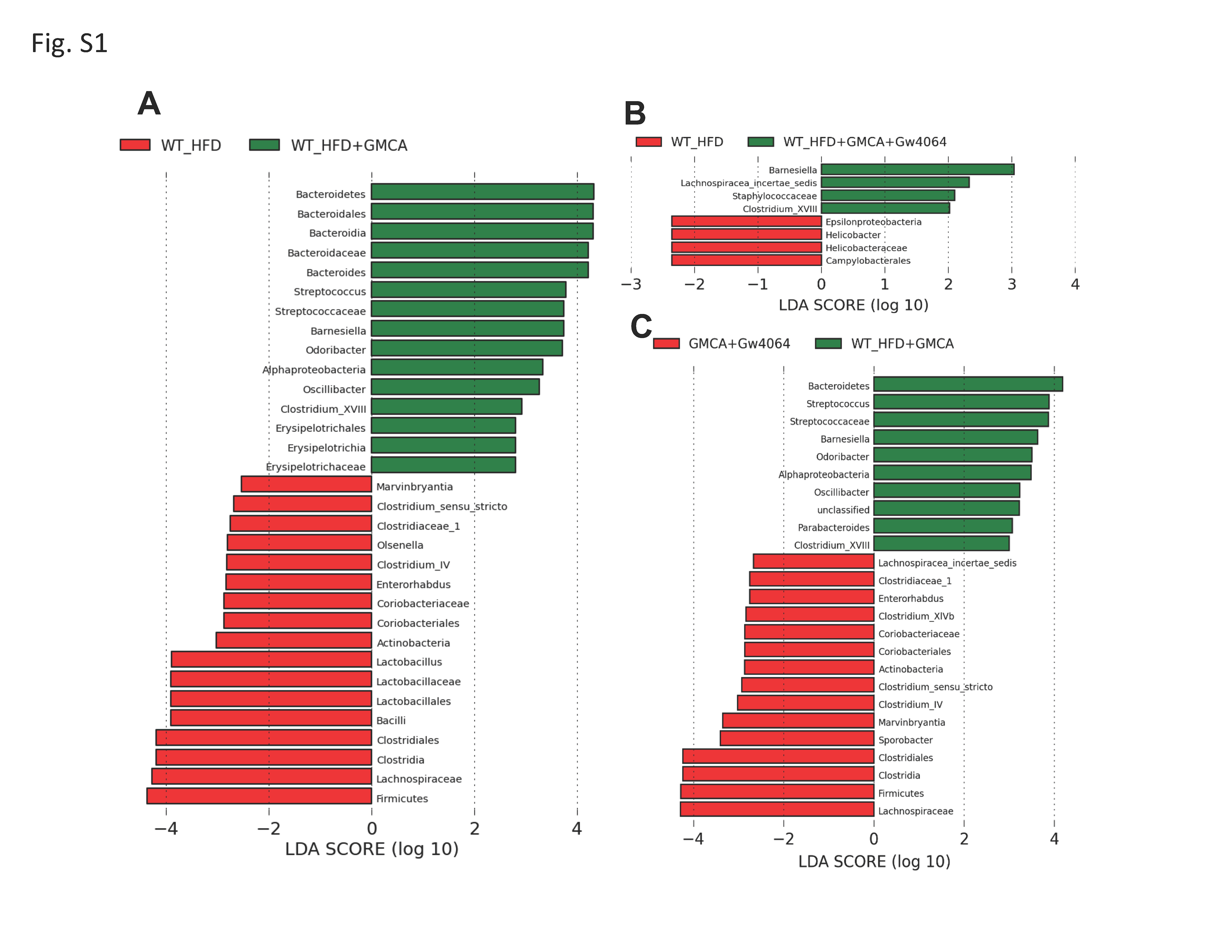

Supplement: Figure S1 [file sys005162056sf1.tif]

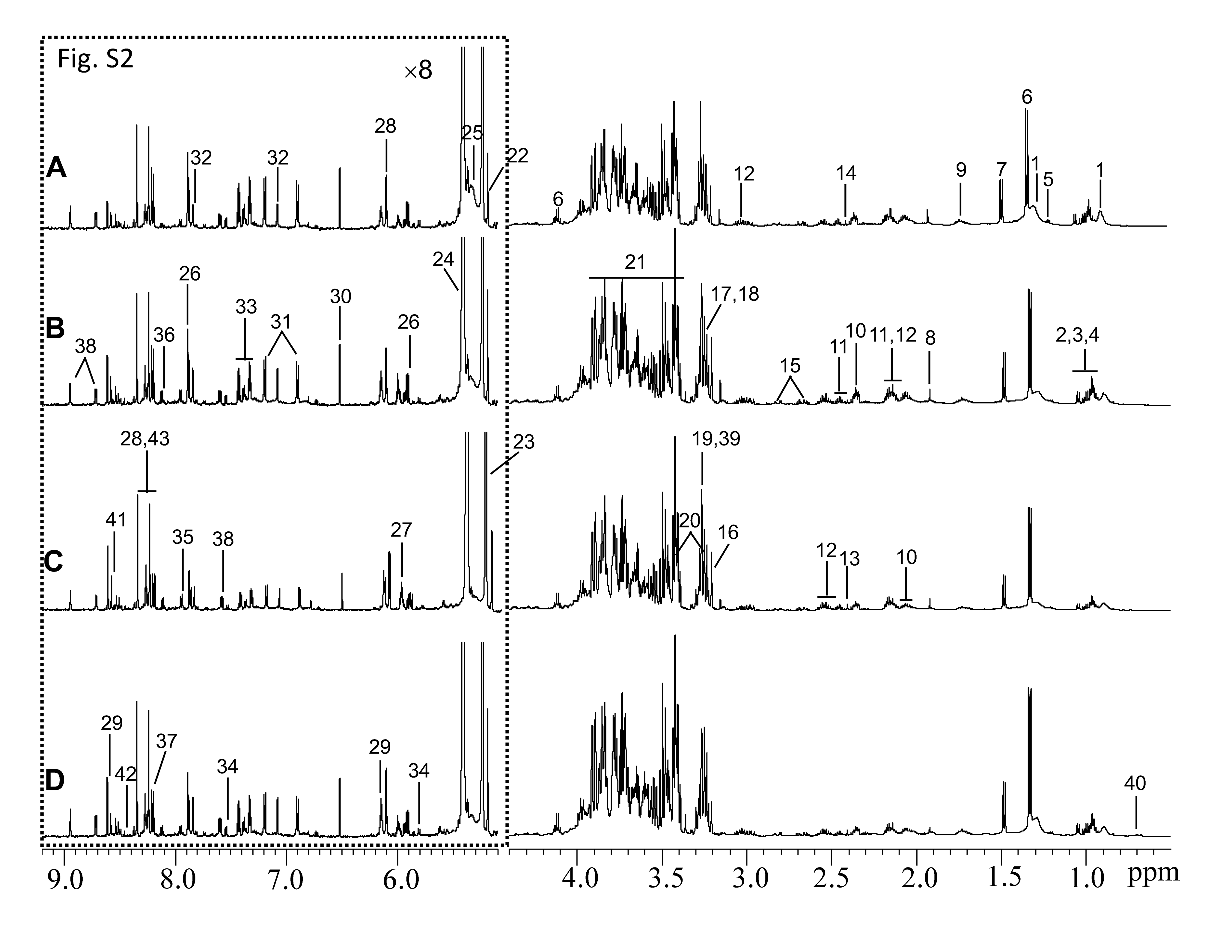

Supplement: Figure S2 [file sys005162056sf2.tif]

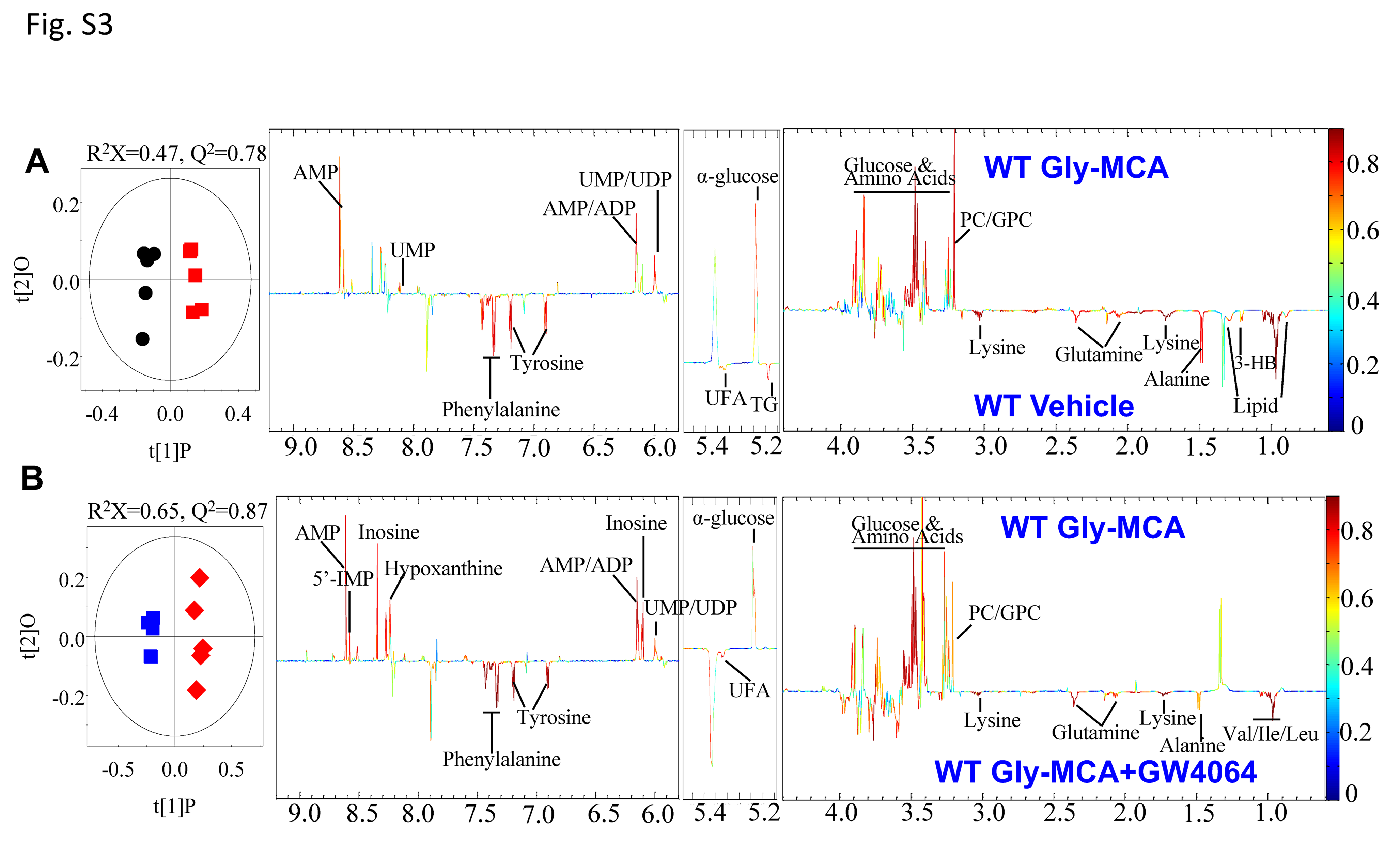

Supplement: Figure S3 [file sys005162056sf3.tif]

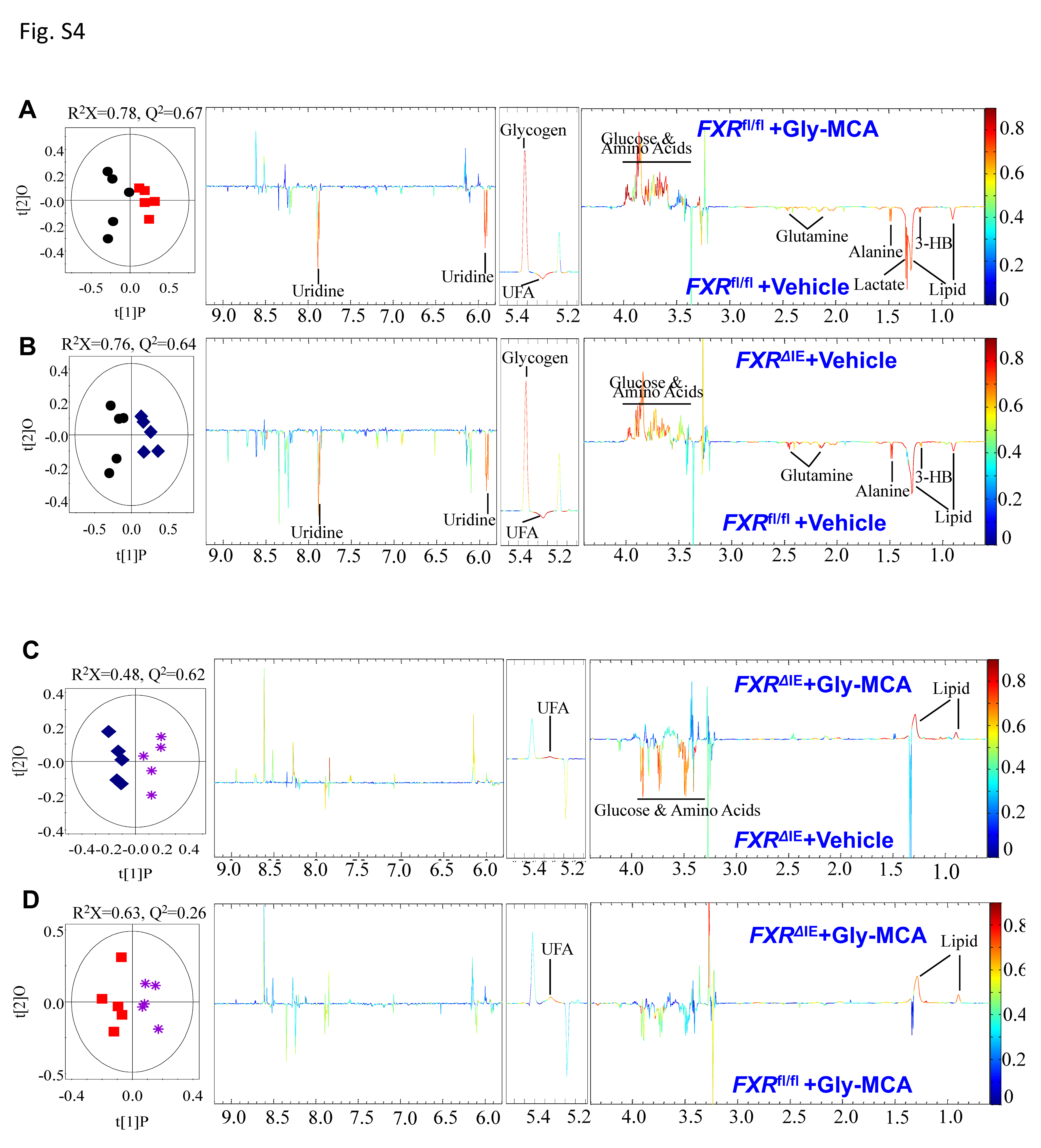

Supplement: Figure S4 [file sys005162056sf4.tif]

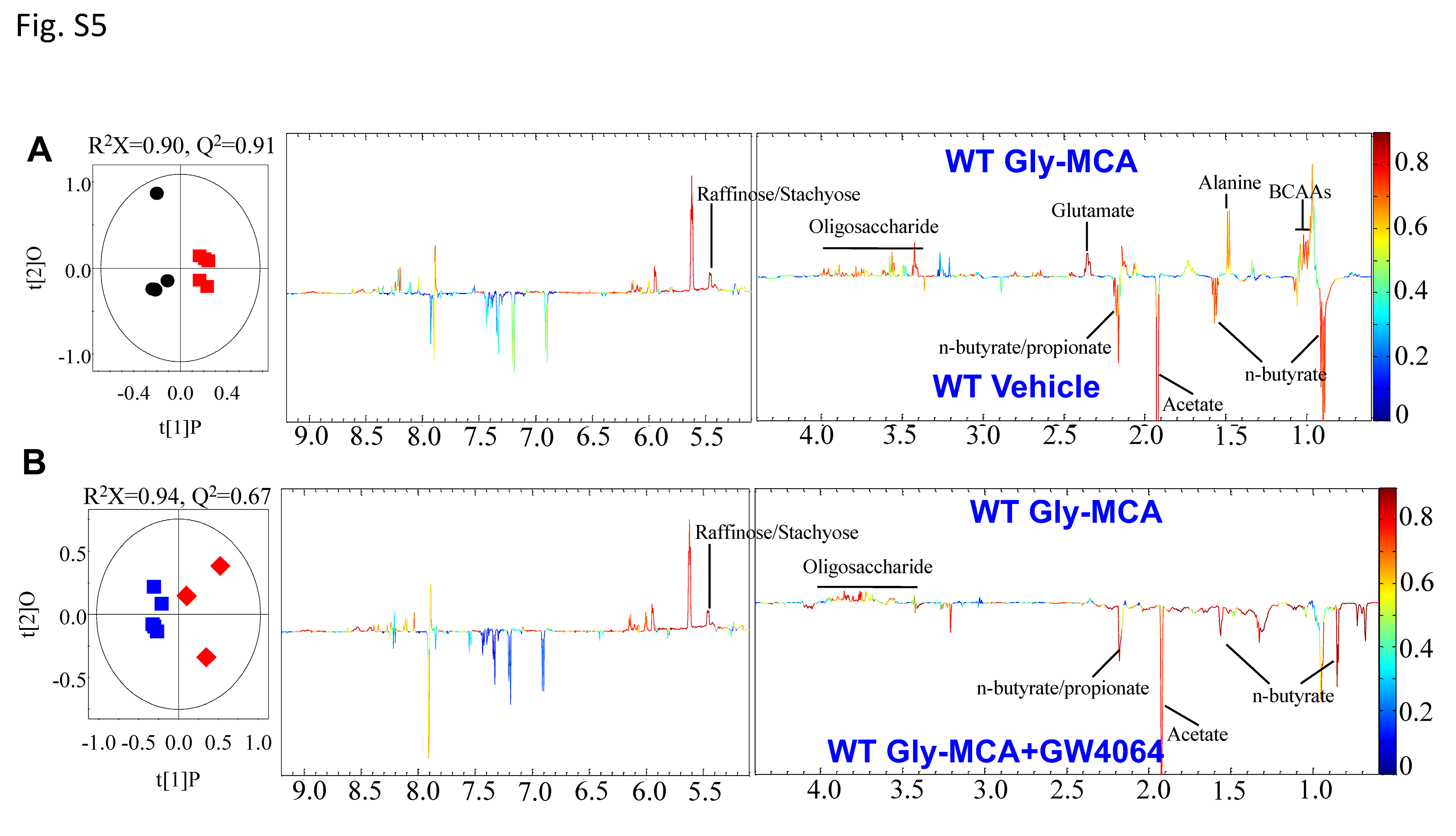

Supplement: Figure S5 [file sys005162056sf5.tif]

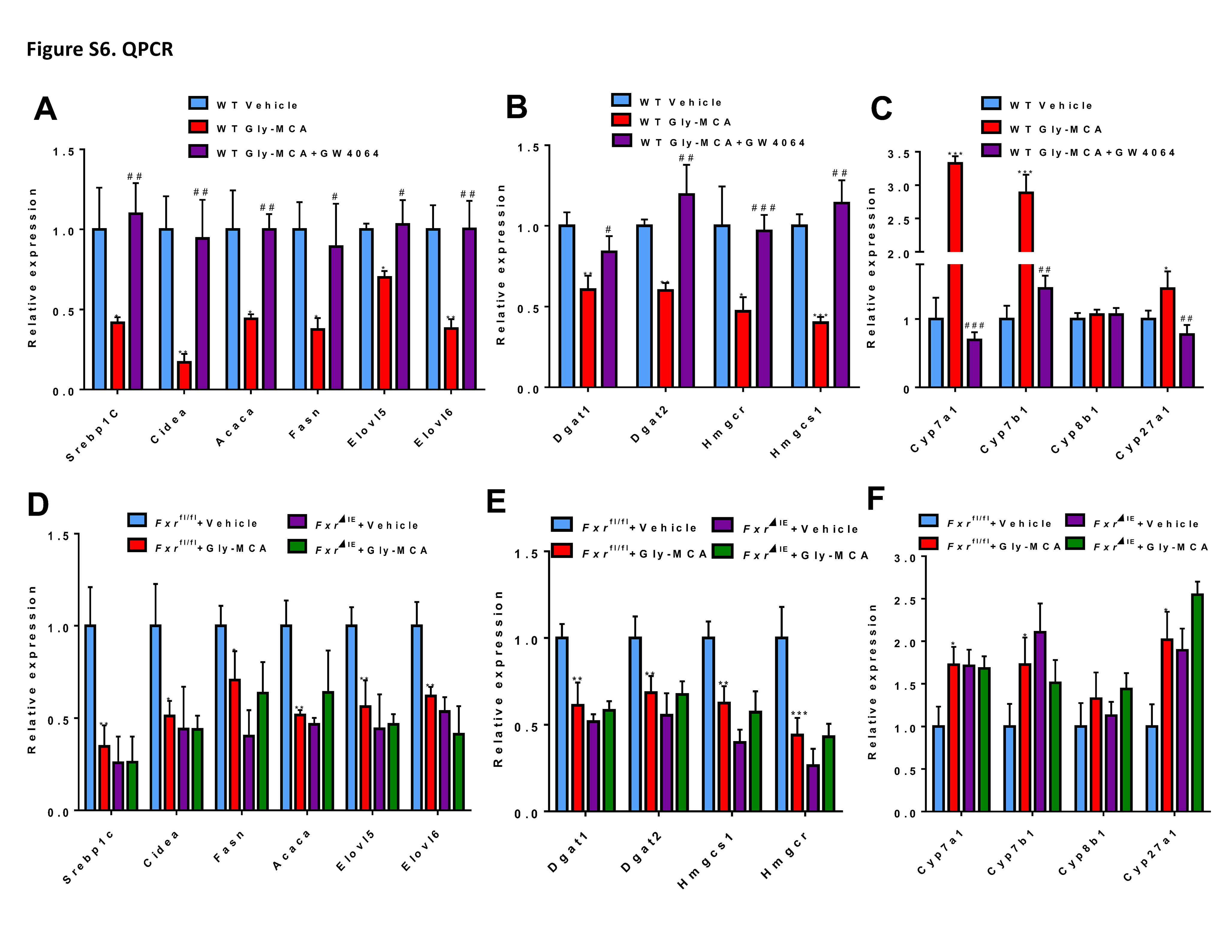

Supplement: Figure S6 [file sys005162056sf6.tif]

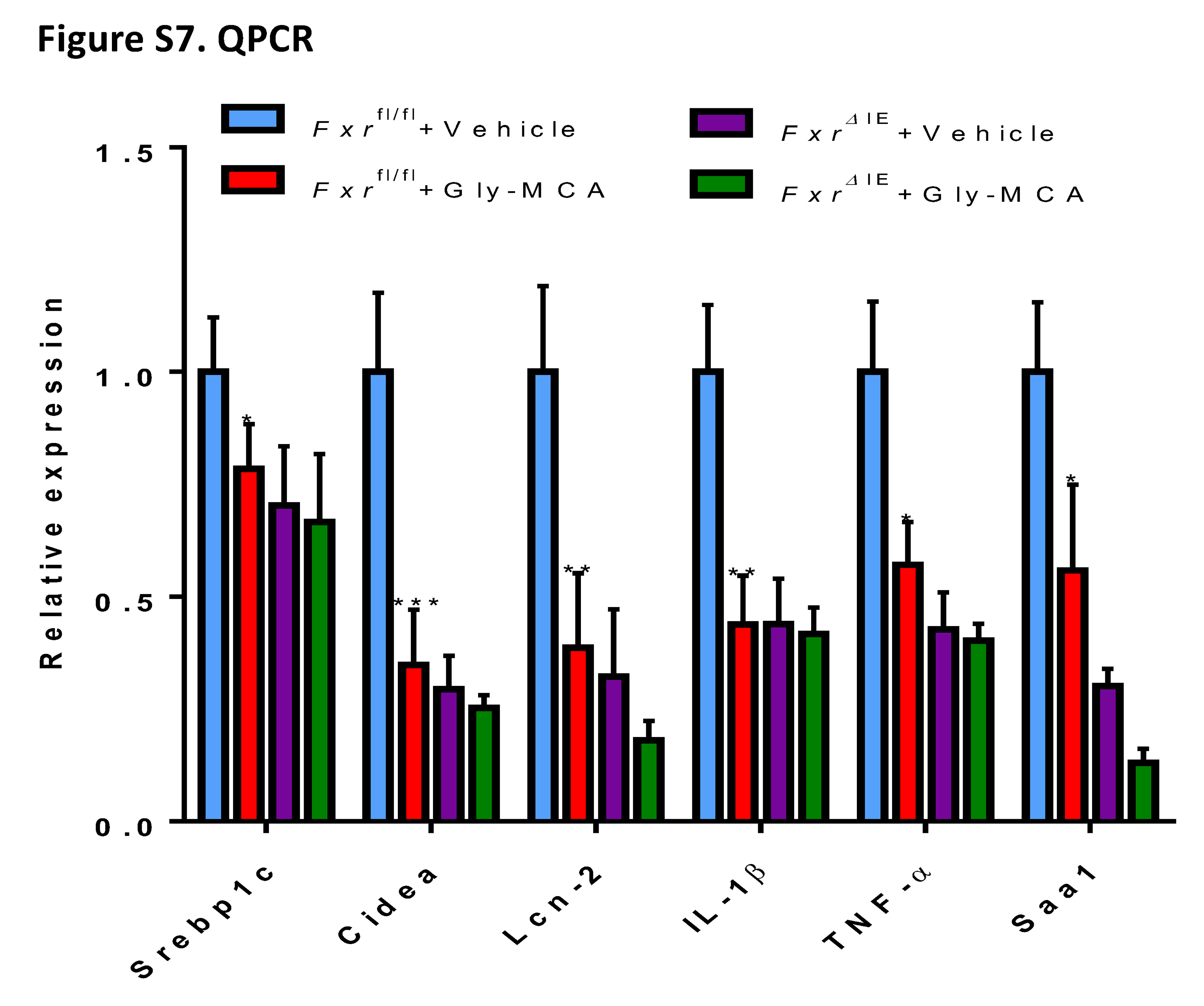

Supplement: Figure S7 [file sys005162056sf7.tif]
