# Supplementary material for: Farnesoid X Receptor Signaling Shapes the Gut Microbiota and Controls Hepatic Lipid Metabolism
Source: mSystems. 2016 Oct 11;1(5):e00070-16. doi: 10.1128/mSystems.00070-16 (PMC5080402; doi:10.1128/mSystems.00070-16)
Supplement: Table S1 [file sys005162056st8.docx]

**Table S1.**

| **Gene** | **Abbreviation** | **Sequence** |
| --- | --- | --- |
| Sterol regulatory element binding  protein-1c | *Srebp1c* | GGAGCCATGGATTGCACATT  GCTTCCAGAGAGGAGGCCAG |
| Cell death-inducing DNA  fragmentation factor, alpha  subunit-like effector A | *Cidea* | TGACATTCATGGGATTGCAGAC  GGCCAGTTGTGATGACTAAGAC |
| Elongation of very long-chain  fatty acids protein 5 | *Elovl5* | CTGAGTGACGCATCGAAATG  CTTGCACATCCTCCTGCTC |
| Elongation of very long-chain  fatty acids protein 6 | *Elovl6* | GAAAAGCAGTTCAACGAGAACG  AGATGCCGACCACCAAAGATA |
| Fatty acid synthase | *Fasn* | AAGGACCTGTCTAGGTTTGATGC  TGGCTTCATAGGTGACTTCCA |
| Acetyl-CoA carboxylase 1 | *Acaca* | ATGTCTGGCTTGCACCTAGTA  CCCCAAAGCGAGTAACAAATTCT |
| Diacylglycerol O-acyltransferase 1 | *Dgat1* | GACGGCTACTGGGATCTGA  TCACCACACACCAATTCAGG |
| Diacylglycerol O-acyltransferase 2 | *Dgat2* | CGCAGCGAAAACAAGAATAA  GAAGATGTCTTGGAGGGCTG |
| Cytochrome P450, family 7,  subfamily A, polypeptide 1  (Cholesterol 7α-hydroxylase) | *Cyp7a1* | AGCAACTAAACAACCTGCCAGT  ACTAGTCCGGATATTCAAGGATGCA |
| Cytochrome P450, family 7,  subfamily B, polypeptide 1  (Oxysterol 7α-hydroxylase) | *Cyp7b1* | TAGCCCTCTTTCCTCCACTCATA  GAACCGATCGAACCTAAATTCCT |
| Cytochrome P450, family 8,  subfamily B, polypeptide 1  (Sterol 12α-hydroxylase) | *Cyp8b1* | GGCTGGCTTCCTGAGCTTATT  ACTTCCTGAACAGCTCATCGG |
| Lipocalin-2 | *Lcn-2* | ATTTCCCAGAGTGAACTGGC  AATGTCACCTCCATCCTGGT |
| Interleukin-1 beta | *IL-1β* | GGTCAAAGGTTTGGAAGCAG  TGTGAAATGCCACCTTTTGA |
| Tumor necrosis factor alpha | *TNF-α* | AGGCTGCCCCGACTACGT  GACTTTCTCCTGGTATGAGATAGCAAA |
| Serum amyloid A 1 | *Saa1* | TCATGTCAGTGTAGGCTCGC  GTCTTCTGCTCCCTGCTCC |
| Serum amyloid A 3 | *Saa3* | AGTAGGCTCGCCACATGTCT  TCCATTGCCATCATTCTTTG |
| 3-hydroxy-3-methyl-glutaryl-CoA  reductase | *Hmgcr* | CACAATAACTTCCCAGGGGT GGCCTCCATTGAGATCCG |
| 3-Hydroxy-3-methylglutaryl-CoA  synthase 1 | *Hmgcs1* | TTCAAAGGAAGTGACCCAGG  GGTCTGATCCCCTTTGGTG |
| Acetyl-Coenzyme A acyltransferase 1B | *Acaa1b* | GAGATGTCTCCCAGCTGCTC  GATGGGATCCTGCCGTCG |
| 3-hydroxy-3-methylglutaryl-CoA  synthase 2 | *Hmgcs2* | TCATTGAACATCAACCGAGC GAAACAACCAGCCTTTCACC |
