# Supplementary material for: Farnesoid X Receptor Signaling Shapes the Gut Microbiota and Controls Hepatic Lipid Metabolism
Source: mSystems. 2016 Oct 11;1(5):e00070-16. doi: 10.1128/mSystems.00070-16 (PMC5080402; doi:10.1128/mSystems.00070-16)
Supplement: Table S2 [file sys005162056st9.docx]

**Table S2.**

| **Key** | **Metabolites** | **Moieties** | **δ ^1^H (ppm) and multiplicity^a^** |
| --- | --- | --- | --- |
| 1 | Lipid | CH_3_, (CH_2_)_n_, CH_2_-C=C, CH_2_-C=O,C-CH_2_-C=,-CH=CH- | 0.89(m), 1.27(m), 2.0(m),  2.3(m), 2.78(m), 5.3(m) |
| 2 | Isoleucine | αCH, βCH, γCH_3_, δCH_3_ | 3.65(d), 1.95(m), 0.99(t), 1.02(d) |
| 3 | Leucine | αCH, βCH_2_, γCH_3_, δCH_3_ | 0.94(d), 3.72(t), 1.96(m), 0.91(d) |
| 4 | Valine | αCH, βCH, γCH_3_ | 3.6(d), 2.26(m), 0.98(d), 1.04(d) |
| 5 | D-3-hydroxybutyrate | CH, CH_2_, γCH_3_, CH_2_ | 4.16(dt),2.41(dd),1.20(d),2.31(dd) |
| 6 | Lactate | αCH, βCH_3_ | 4.11(q), 1.32(d) |
| 7 | Alanine | αCH, βCH_3_ | 3.77(q), 1.48(d) |
| 8  9  10  11 | Acetate  Lysine  Glutamate Glutamine | CH3  αCH, βCH, γCH3  αCH, βCH2, γCH2  αCH, βCH2, γCH2 | 1.91(s)  3.74(t), 1.90(m), 1.72(m)  2.08(m), 2.34(m), 3.75(m)  2.15(m), 2.44(m), 3.77(m) |
| 12 | Glutathione | CH_2_, CH_2_, S-CH_2_, N-CH, CH | 2.16(m), 2.55(m), 2.95(dd), 3.78(m), 4.56(q) |
| 13 | Succinate | CH_3_ | 2.41(s) |
| 14 | Pyruvate | CH_3_ | 2.38(s) |
| 15  16  17 | Aspartate  Choline  Phosphocholine(PC) | αCH, βCH2, γCH2  N(CH_3_)_3_, OCH_2_, NCH_2_  N(CH_3_)_3_, OCH_2_, NCH_2_ | 3.90(m), 2.68(m), 2.82(dd)  3.2(s), 4.05(t), 3.51(t)  3.22(s), 4.21(t), 3.61(t) |
| 18 | Glycerophosphocholine | N(CH_3_)_3_, OCH_2_, NCH_2_ | 3.22(s), 4.32(t), 3.68(t) |
| 19 | TMAO | CH_3_ | 3.27(s) |
| 20 | Taurine | S-CH_2_, N-CH_2_ | 3.26(t), 3.40(t) |
| 21 | Glucose & amino acids | αCH resonances | 3.3-3.9 |
| 22 | Triglycerides | CH | 4.08(m), 4.21(m), 5.18(m) |
| 23 | α-Glucose | 1-CH | 5.23(d) |
| 24 | Glycogen | 1-CH | 5.38-5.45(m) |
| 25 | Unsaturated fatty acid | CH=CH | 5.3(m) |
| 26 | Uridine | 11-CH, 7-CH, 12-CH, 6-CH, 5-CH, 4-CH, CH_2_, CH_2_ | 7.88(d), 5.92(d), 5.9(d), 4.36(m), 4.24(t) |
| 27 | Uridine diphosphate (UDP) | C6,ring, C1’H,ribose  C5,ring, C3’H,ribose  C5’H,ribose, 4’H,ribose | 7.94(d)  5.98(d)  5.97(d) |
| 28 | Inosine | 14-CH, 1-CH, 8-CH, 4’-CH, 5’-CH, CH_2_(1/2), CH_2_(1/2) | 8.34(s), 6.09(d), 8.24(s), 4.76(t),  4.47(m) |
| 29 | Adenosine monophosphate (AMP) | 2-H, 8-H, 2’-H | 8.61(s), 8.27(s), 6.15(d) |
| 30 | Fumarate | CH | 6.53(s) |
| 31 | Tyrosine | CH, CH | 6.89(dd), 7.18(dd) |
| 32 | Histidine | 2-CH, 4-CH, CH_2_ | 7.75(t), 7.08(d), 6.05(d) |
| 33 | Phenylalanine | Ring-CH | 7.40(m), 7.33(m), 7.35(m) |
| 34  35 | Uracil  Xanthine | 1-CH, 2-CH  CH | 5.81(d), 7.54(d)  7.88(s) |
| 36 | Uridine monophosphate (UMP) | C6,ring, C1’H,ribose  C5,ring, C3’H,ribose  C5’H,ribose, 4’H,ribose | 8.12(d)  5.98(d)  5.97(d) |
| 37 | Hypoxanthine | CH, CH | 8.20(s), 8.21(s) |
| 38 | Nicotinamide | 2-CH, 6-CH, 4-CH, 5-CH | 8.93(s),8.62(d), 8.25(d),7.60(dd), |
| 39 | Betaine | CH_2_, CH_3_ | 3.27(s), 3.93(s) |
| 40 | Bile acid | CH_3_ | 0.73(m) |
| 41 | Inosine-5’-monophosphate (5’-IMP) | 2-H, 8-H, 2’-H | 8.54(s), 8.28(s), 6.15(d) |
| 42 | Formate | CH | 8.45(s) |
| 43 | Adenosine | 14-CH | 8.34(s) |

^a^ Key: s, singlet; d, doublet; t, triplet; q, quartet; m, multiplet; dd, doublet of doublet.
