# Supplementary material for: Farnesoid X Receptor Signaling Shapes the Gut Microbiota and Controls Hepatic Lipid Metabolism
Source: mSystems. 2016 Oct 11;1(5):e00070-16. doi: 10.1128/mSystems.00070-16 (PMC5080402; doi:10.1128/mSystems.00070-16)
Supplement: Table S3 [file sys005162056st10.docx]

**Table S3.**

| Species | Phylum | Reference |
| --- | --- | --- |
| *Bacteroidesthetaiotamicron* | Bacteroidetes | 1, 2 |
| *Porphyromonasgingivalis* | Bacteroidetes | 3 |
| *Faecalibacteriumprausnitzii* | Firmicutes | 4 |
| *Eubacteriumrectale* | Firmicutes | 5 |
| *Streptococcus thermophilus* | Firmicutes | 1, 6 |
| *Enterococcus faecalis* | Firmicutes | 7 |
| *Lactobacillus casei* | Firmicutes | 8 |
| *Bifidobacterium adolescentis* | Actinobacteria | 9 |
| *Escherichia coli* | Proteobacteria | 10 |
| *Klebsiella pneumonia* | Proteobacteria | 2, 11 |

**REFERENCES**

1. **Heinken A, Sahoo S, Fleming RMT, Thiele I.**2013. Systems-level characterization of a hostmicrobe metabolic symbiosis in the mammalian gut. *Gut Microbes* 4(1):28–40.
2. **Heinken A, Thiele I.**2015. Systematic prediction of health-relevant human-microbial

co-metabolism through a computational framework. *Gut Microbes* 6(2):120–130.

3. **Mazumdar V, Snitkin ES, Amar S, Segre D.** 2009. Metabolic Network Model of a Human Oral Pathogen. *J Bacteriol* 191(1):74–90.

4. **Heinken A, Khan MT,Paglia G, Rodionov DA, Harmsen HJM, Thiele I.** 2014. Functional metabolic map of Faecalibacterium prausnitzii, a beneficial human gut microbe. *J Bacteriol* 196(18):3289–3302.

5. **Shoaie S, Karlsson F, Mardinoglu A, Nookaew I, Bordel S, Nielsen J.** 2013. Understanding the interactions between bacteria in the human gut through metabolic modeling. *Sci Rep* 3:2532.

6. **Pastink MI, Teusink B, Hols P, Visser S, de Vos WM, Hugenholtz J.** 2009. Genome-scale model of Streptococcus thermophilus LMG18311 for metabolic comparison of lactic acid bacteria. *Appl Environ Microbiol* 75(11):3627–33.

7. **Veith N, Solheim M, van Grinsven KWA, Olivier BG, Levering J, Grosseholz R, Hugenholtz J, Holo H, Nes I, Teusink B, Kumme U.** 2015. Using a genome-scale metabolic model of Enterococcus faecalis V583 to assess amino acid uptake and its impact on central metabolism. *Appl Environ Microbiol* 81(5):1622–1633.

8. **Vinay LE, Hamilton JJ, Stahl B, Broadbent JR, Reed JL, Steele JL.** 2014 Genome -scale reconstruction of metabolic networks of lactobacillus casei ATCC 334 and 12A. *PLoS One* 9(11). doi:10.1371/journal.pone.0110785.

9. **El-Semman IE, Karlsson FH, Shoaie S, Nookaew I, Soliman TH, Nielsen J.** 2014. Genome-scale metabolic reconstructions of Bifidobacterium adolescentis L2-32 and Faecalibacterium prausnitzii A2-165 and their interaction. *BMC Syst Biol* 8(1):41.

10. **Orth JD, Conrad TM, Na J, Lerman JA, Nam H, Feist AM, Palsson B.** 2014. A comprehensive genome-scale reconstruction of Escherichia coli metabolism-2011. *Mol Syst Biol* 7(1):535–535.

11. **Liao Y-C, Huang TW, Chen FC, Charusanti P, Hong JSJ, Chang HY, Tsai SF, Palsson B, Hsiung CA. 2011.** An experimentally validated genome-scale metabolic reconstruction of Klebsiella pneumoniae MGH 78578, iYL1228. *J Bacteriol* 193(7):1710–1717.
